# Supplementary material for: Undecidability in quantum thermalization
Source: Nat Commun. 2021 Aug 24;12:5084. doi: 10.1038/s41467-021-25053-0 (PMC8384856; doi:10.1038/s41467-021-25053-0)
Supplement: Supplementary file 1 — Supplementary Information [file 41467_2021_25053_MOESM1_ESM.pdf]

Supplementary Note for “Undecidability in quantum thermalization”

Naoto Shiraishi*and Keiji Matsumoto†

Contents

1 Introduction **2**

2 Background of quantum thermalization **2**

3 Background of theoretical computer science **3**

4 Setup and main results **5**

5 Proof of Theorem 1a and Theorem 1b (undecidability of relaxation) from Lemma 1 **9**

6 Strategy for the proof of Lemma 1 **10**

7 Proof of Lemma 1: (1) Classical Turing machine **11**

7.1 Universal reversible Turing machine 11

7.2 Generalized URTM 13

7.3 The states, symbols, and tape of M_{G} 13

7.4 The move of M_{G}: the first step 15

7.5 The move of M_{G}: simulating TM1 and TM2 15

7.6 The move of M_{G}: simulating TM3 15

8 Proof of Lemma 1: (2) Quantum partial isometry corresponding to M_{G} **16**

8.1 Toy example of Feynman-Kitaev type Hamiltonian 16

8.2 Construction of quantum partial isometry for M_{G} 17

9 Proof of Lemma 1: (3) Evaluating $\overline{\mathcal{A}}$ for computational basis state **19**

9.1 General expression of long-time average 19

9.2 Hamiltonian emulating M_{G} and effective Hamiltonian 19

9.3 Computing long-time average of $\mathcal{A}$ 20

10 Proof of Lemma 1: (4) Evaluating $\overline{\mathcal{A}}$ for superposition of computational basis states **21**

10.1 Setting of the initial state and decoding of the input 21

*Department of physics, Gakushuin university, 1-5-1 Mejiro, Toshima-ku, Tokyo, 171-8588, Japan

†Quantum Computation Group, National Institute of Informatics, 2-1-2 Hitotsubashi, Chiyoda-ku, Tokyo 101-8430, Japan

10.2 Case when TM2 halts 1: decoding and expression of states 23
10.3 Case when TM2 halts 2: long-time average of $\mathcal{A}$ 25
10.4 Case when TM2 does not halt 27
10.5 Note: dimension of the local Hilbert space 28

11 Proof of Theorem 2 (undecidability of thermalization) 29
11.1 Value of $\overline{\mathcal{A}}$ in thermodynamic limit when TM2 halts 29
11.2 Pushing the value of $\overline{\mathcal{A}}$ away from zero 30
11.3 Tuning the microcanonical average 32

12 Remarks on the infinite limit 34
12.1 What happens if we numerically simulate this system? 34
12.2 Difference from the behavior of near-integrable systems 35

1 Introduction

This Supplementary Note aims to provide the full proof of the theorem and lemma in the main text (which is called Theorem 1b, Theorem 2, and Lemma 1 in this Supplementary Note). The following two tools inspire our proof: the reduction to the halting problem of Turing machines [1,2] and Feynman-Kitaev type quantum emulation of classical machines [3, 4]. Combining these ideas properly, we successfully construct a quantum many-body system where the halting problem of Turing machines determines the destination after relaxation.

This Supplementary Note is organized as follows: In Sec. 2 and Sec. 3, we provide a pedagogical review of quantum thermalization and theoretical computer science. In Sec. 4, we state three main theorems and a technical lemma. The latter lemma is the most important result from a theoretical aspect. In Sec. 5, we derive two theorems, undecidability of relaxation, from the technical lemma. Most of the remainder of this Supplementary Note is devoted to proving this lemma. In Sec. 6, we briefly sketch the proof strategy. In Sec. 7, we introduce a classical universal reversible Turing machine, which is responsible for the halting problem. In Sec. 8, we construct the Hamiltonian of the quantum system emulating the classical dynamics of the Turing machine. Since the details of dynamics of our quantum system are a little complicated, in Sec. 9 we introduce an analogous setting, which is easier to treat, and solve the dynamics. In Sec. 10, we go back to the original setting and construct the initial state with which the expectation value after relaxation is indeed undecidable. In Sec. 11, we prove the remaining theorem, undecidability of thermalization, by extending the proof techniques for the previous lemma. In Sec. 12, we briefly comment on what happens if we numerically simulate the constructed system.

2 Background of quantum thermalization

Before going to our main result, we first summarize the problem of thermalization and theoretical computer science in this and next sections for readers who are not familiar with these topics. If a reader is familiar with them, one can skip this and the next sections.

In the research field of quantum thermalization, we mainly consider whether a quantum many-body system with a Hamiltonian H at the initial state $|\psi\rangle$ thermalizes (with respect

to an observable $\mathcal{A}$) or not. A state $|\phi\rangle$ is called *thermal* with respect to an observable $\mathcal{A}$ if its expectation value of $\mathcal{A}$ is close to its equilibrium value:

$$\langle\phi|\mathcal{A}|\phi\rangle \simeq \mathrm{Tr}[\mathcal{A}\rho^{\mathrm{MC}}], \tag{S.1}$$

where ρ^{MC} is a microcanonical state with energy $\langle\phi|H|\phi\rangle$. The symbol $\simeq$ means that both-hand sides coincide in the thermodynamic limit (i.e., If $\mathcal{A} = O(1)$, $\mathcal{A} \simeq \mathcal{B}$ means $\lim_{V\to\infty}(\mathcal{A} - \mathcal{B}) = 0$, and if $\mathcal{A} = O(V)$, $\mathcal{A} \simeq \mathcal{B}$ means $\lim_{V\to\infty}\left(\frac{\mathcal{A}}{V} - \frac{\mathcal{B}}{V}\right) = 0$). We call that an initial state $|\psi\rangle$ under the Hamiltonian H *thermalizes* with respect to $\mathcal{A} = O(1)$ [1] if $|\psi(t)\rangle := e^{-iHt}|\psi\rangle$ is thermal with respect to $\mathcal{A}$ for almost all t, that is,

$$\lim_{V\to\infty}\lim_{T\to\infty}\frac{1}{T}\int_0^T dt\chi\{\left|\langle\psi(t)|\mathcal{A}|\psi(t)\rangle - \mathrm{Tr}[\mathcal{A}\rho^{\mathrm{MC}}]\right| < \varepsilon\} = 1 \tag{S.2}$$

is satisfied for any $\varepsilon > 0$. Here $\chi\{\cdot\}$ is the indicator function which takes 1 (resp. 0) if the statement inside the bracket is true (resp. false). In the above case, $\chi\{\cdot\}$ takes one if $\left|\langle\psi(t)|\mathcal{A}|\psi(t)\rangle - \mathrm{Tr}[\mathcal{A}\rho^{\mathrm{MC}}]\right| < \varepsilon$ is satisfied at the time t, and takes zero otherwise. Note that due to the quantum recurrence theorem [5], for any T' there exists $\tau > T'$ such that the state at time τ, $|\psi(\tau)\rangle$, and the initial state $|\psi(0)\rangle$ is arbitrarily close. Our definition of thermalization allows recurrence, while recurrence time should become extremely long in a large system.

Let $\bar{\mathcal{A}} := \lim_{T\to\infty}\frac{1}{T}\int_0^T dt\,\langle\psi(t)|\mathcal{A}|\psi(t)\rangle$ be the long-time average of $\mathcal{A}$. Then, an initial state thermalizes if the following two conditions are satisfied:

- (Relaxation): The time-series fluctuation around the long-time average $\bar{\mathcal{A}}$ converges to zero:
$$\lim_{T\to\infty}\frac{1}{T}\int_0^T dt(\langle\psi(t)|\mathcal{A}|\psi(t)\rangle - \bar{\mathcal{A}})^2 \simeq 0. \tag{S.3}$$
- (Convergence to the equilibrium value): The long-time average $\bar{\mathcal{A}}$ converges to the equilibrium value $\mathrm{Tr}[\mathcal{A}\rho^{\mathrm{MC}}]$:
$$\overline{\mathcal{A}} \simeq \mathrm{Tr}[\mathcal{A}\rho^{\mathrm{MC}}]. \tag{S.4}$$

The former condition, relaxation, is proven for initial states under some condition (a diverging effective dimension) [6–8] and this condition is shown to be fulfilled analytically in physically plausible initial states [9]. Thus, the remaining hard task is to handle the latter condition, convergence to the equilibrium value. This is why various concepts and arguments raised in the field of quantum thermalization concern the long-time average and the equilibrium value.

The main goal of this Supplementary Note is to prove the incomputability of the value of the long-time average $\overline{\mathcal{A}}$. In other words, we have no general procedure to detect the behavior of quantum many-body systems after relaxation.

3 Background of theoretical computer science

We here briefly review some basic notions of theoretical computer science; the Turing machines, the Church-Turing thesis, decision problems, and the halting problem of Turing

[1] It is easy to extend the definition to the case of $\mathcal{A} = O(V)$.

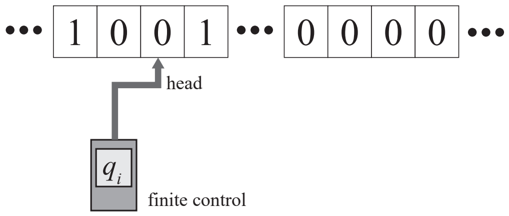

Supplementary Figure 1: Schematic of a Turing machine (TM). The finite control with the internal state q_i reads the cell with its symbol 0.

machines. Detailed explanations can be seen in e.g., the textbook of Moore and Mertens [2].

We employ the description with Turing machines (TM) as computation. The TM is a very simple computation system which consists of a one-dimensional *tape* (an infinite line of cells) filled with symbols and a *finite control* with a *head* and its own *internal state* (see Supplementary Figure 1). The head reads a single cell on the tape, and can rewrite the symbol of the cell, and can move left or right one cell. At the same time, the internal state of the finite control may change. The *transition function* specify the rule of dynamics in the following manner as an example:

> If the internal state of the finite control is q_i and the symbol in the cell read by the head is g_m, then the machine changes the internal state to q_j, rewrites the cell to g_n, and moves the head left.

A TM is defined as a set of tape alphabets, a set of internal states, and a transition function[2]. One may feel that TMs are very primitive and too simple. However, there exist universal Turing machines which can implement all possible TMs, and these universal Turing machines can implement almost all computational tasks in our world (e.g., computation by C++ and Python). Thus, it is declared that computational functions are those computable by TMs, which is known as the Church-Turing thesis. By accepting the Church-Turing thesis, our computational ability is equivalent to that of a TM, and therefore we can safely restrict our attention to a universal Turing machine.

We next explain *decision problems*. In decision problems, a set of infinite inputs with a Yes/No assignment is given, and our task is to answer Yes/No correctly for all inputs. In the case of the primary test, for example, inputs are natural numbers, and we need to answer Yes if and only if the input is a prime number. If there exists a TM which outputs 1 with any input assigned to Yes and outputs 0 with any input assigned to No, this decision problem is called *decidable*. If a decision problem is not decidable (i.e., no TM answers this problem correctly), this decision problem is called *undecidable*. We note that the speed of computation does not matter to decidability. In fact, combinatorial optimization problems, which are regarded as hard tasks in practice, are categorized as decidable problems because we can solve them by brute force methods. We also note that undecidable problems must accompany infinite possible inputs because there always exists a TM whose output coincides with the correct answer for finite inputs accidentally. This is one of the reasons why standard setups of theoretical computer science usually accompany infinite inputs.

[2]Precisely, we also require that the tape alphabet should contain a blank cell, the set of internal state should contain a start state and a halting state

A famous undecidable problem is *the halting problem of TMs.* In this decision problem, inputs are input codes for a fixed universal Turing machine[3], and we need to answer whether this TM halts at some time or moves forever. It is proved that this decision problem is undecidable. In our proof of the undecidability of thermalization, we reduce our problem to the halting problem of TMs.

We sometimes treat decision problems *with promise.* In the case with promise, the inputs are restricted to a subclass of possible inputs where this promise is satisfied. In other words, we allow not to answer or to answer incorrectly if the input does not satisfy this promise. We again take the primary test as an example. Suppose a promise that the input is a prime or a product of two primes. Then, if the input is a product of three primes, we may incorrectly answer Yes (a TM may output 1) for this input.

4 Setup and main results

We first state the undecidability of *relaxation* and then state the undecidability of *thermalization* in this section. All the essential ideas for the proof of the undecidability of thermalization have already appeared in that of relaxation. Therefore, in this Supplementary Note we first treat that of relaxation, which spends most of this Supplementary Note, and then briefly discuss some additional ideas to extend the result of relaxation to thermalization.

Consider a one-dimensional chain of d-level quantum systems with the periodic boundary condition, whose underlying local Hilbert space is denoted by $\mathcal{H}$. Suppose that the Hamiltonian of this chain H is shift-invariant and contains only 1-body terms and nearest-neighbor 2-body interactions. We set the length of the system as L. Let A be a non-negative observable of $\mathcal{H}$, and $\mathcal{A}_L$ be the normalized spatial average of A's:

$$\mathcal{A}_L := \frac{1}{L}\sum_{i=1}^{L} A_i, \tag{S.5}$$

where A_i is the operator A acting on the site i. Our interest is a long-time average of $\mathcal{A}_L$:

$$\overline{\mathcal{A}}(H,\rho) := \lim_{L\to\infty}\lim_{T\to\infty}\frac{1}{LT}\int_0^T \mathrm{Tr}[e^{-iHt}\rho_L e^{iHt}\mathcal{A}_L]dt, \tag{S.6}$$

where ρ_L is the initial state of the system[4] with length[5] L. Whenever without confusion, the dependency of $\overline{\mathcal{A}}$ on H and ρ is dropped.

We argue, roughly, that $\overline{\mathcal{A}}$ is incomputable[6]. Our statement still holds even in the case

[3] Recall that a single universal Turing machine emulates any TM with any input.

[4] More precisely, we first introduce a state ρ with infinite length, and define ρ_L as the restriction of the state ρ to the first L consecutive sites.

[5] We note that how the system size increases (e.g., $1 \le i \le L$ or $-L/2+1 \le i \le L/2$) does not matter to our result of undecidability because this system has the periodic boundary condition and almost uniform initial states in the form of $|\phi_0\rangle\,|\phi_1\rangle^{\otimes L-1}$ suffices to show the undecidability.

[6] To make such an assertion appropriately, some vocabulary from computer science is necessary: We say the operator A is computable if a Turing machine can compute any component (with respect to some standard basis) of A with any specified accuracy (So the inputs of a Turing machine are the indices of the components and the integer specifying the accuracy). Also, the Hamiltonian and the density operator over the one-dimensional chain are computable if their restriction to the finite system size (L) is computable. In our case, the Hamiltonian is computable if and only if its 1- and 2- body terms are computable.

Whenever we say an operator A is an input to the problem, we mean that the bit string describing A is given to a Turing machine as the input. Here, the map of the bit string to an arbitrary component should be computable up to any given accuracy. When the input is an observable or a state over $\mathcal{H}^{\otimes\infty}$, the bit string description is given again. The computability of the map of the bit string to the observable or the

that the Hamiltonian is shift-invariant and nearest-neighbor interaction, and the initial state is an almost uniform product state in the following form:

$$\rho_L = |\phi_0\rangle\,|\phi_1\rangle^{\otimes L-1}\,. \tag{S.7}$$

To state our claim in a rigorous manner, we define two decision problems with a promise; STA (state time average) and HTA (Hamiltonian time average).

[**STA**]: The dimension of the local Hilbert space $d = \dim \mathcal{H}$, a Hamiltonian H, and a one-body observable A are fixed. These two, H and A, are parameters of the problem[7].

Input: a density operator ρ.

Promise: Either $\overline{\mathcal{A}} \in [c+\varepsilon_1, c-\varepsilon_1]$ or $\overline{\mathcal{A}} \notin [c+\varepsilon_2, c-\varepsilon_2]$ with $0 < \varepsilon_1 < \varepsilon_2$ holds.

Decision problem: Decide which of the above two holds.

[**HTA**]: The dimension of the local Hilbert space $d = \dim \mathcal{H}$, a one-body observable A, and an initial state ρ are fixed. These two, A and ρ, are parameters of the problem[8].

Input: a Hamiltonian H.

Promise: Either $\overline{\mathcal{A}} \in [c+\varepsilon_1, c-\varepsilon_1]$ or $\overline{\mathcal{A}} \notin [c+\varepsilon_2, c-\varepsilon_2]$ with $0 < \varepsilon_1 < \varepsilon_2$ holds.

Decision problem: Decide which of the above two holds.

We now state our main theorems, which claim that these decision problems are undecidable. Below, the necessary dimension d_0 is a fixed number, whose rough estimation is presented in Sec. 10.5 as $d_0 \simeq 120$.

We first state the undecidability of relaxation in the form of STA:

Theorem 1a

Fix the local dimension $d \geq d_0$. Then, there exists a family of shift-invariant Hamiltonians H with 1-body terms and 2-body nearest-neighbor interaction terms having the following properties:
Let A be an arbitrary observable on a single site and $\varepsilon > 0$ be an arbitrary error margin. We put no requirement on the observable.
Then, for any $M > 1$ there exist an operator A' with $\|A - A'\| \leq \varepsilon$, a value A^*, and proper errors ε_1 and ε_2 with $\varepsilon_2 \leq \varepsilon$ and $\varepsilon_2/\varepsilon_1 \geq M$ such that the STA with the aforementioned A', H, $c = A^*$, ε_2 and ε_1 is undecidable. This remains valid even if the initial state ρ (inputs for STA) is restricted to a product state of a pure state of a single site in the form of Eq. (S.7), where $|\phi_0\rangle$ and $|\phi_1\rangle$ are orthogonal to each other.

We here clarify the dependency of quantities: The system Hamiltonian H is fixed independent of the observable A and a small parameter ε. In contrast, the target value A^* and

state is defined considering restriction to the finite-size system.

[7]We also require that H and A are computable.

[8]We also require that A and ρ are computable.

two parameters for errors ε_1 and ε_2 depend on H, A, and the parameters ε and M. The task of STA is to determine $\overline{\mathcal{A}} \in [c+\varepsilon_1, c-\varepsilon_1]$ or $\overline{\mathcal{A}} \notin [c+\varepsilon_2, c-\varepsilon_2]$ with $c = A^*$ for all inputs ρ with the form (S.7), and thus to show undecidability we pick up a family of *bad* inputs depending on A, ε_1, and ε_2.

We slightly modified the observable from A to A' in order to avoid several unwanted cases that the observable A is an identity operator and that the basis determined by the Hamiltonian H and the eigenbasis of A are accidentally aligned in an unwanted direction.

We next state the undecidability of relaxation in the form of HTA. The next theorem on HTA, Theorem 1b, is stronger than Theorem 1a in that two of three main quantities, the initial state, the observable, and the Hamiltonian, are arbitrary.

Theorem 1b

Fix the local dimension $d \geq d_0$. Let A be an arbitrary observable on a single site and ρ be an arbitrary pure state in the form of Eq. (S.7). We require that there exists a state $|\phi_2\rangle$ orthogonal to $|\phi_0\rangle$ and $|\phi_1\rangle$ such that $\langle\phi_2|A|\phi_2\rangle \neq \langle\phi_1|A|\phi_1\rangle$.
Then, for any large $M > 1$, there exist proper ε_2, ε_1 with $\varepsilon_2/\varepsilon_1 \geq M$ and A^* such that the HTA for A and ρ with $c = A^*$, ε_2 and ε_1, is undecidable. This remains valid even if the Hamiltonian H (inputs of HTA) is restricted to a shift-invariant Hamiltonian H with 1-body terms and 2-body nearest-neighbor interaction terms.

Here, we put the assumption on $|\phi_2\rangle$ in order to exclude the case that A is close to an identity operator.

We again clarify the dependency of quantities: Both the observable A and the initial state ρ are arbitrary (as far as the condition for $|\phi_2\rangle$ is satisfied). Two parameters for the error, ε_1 and ε_2, depend on the choice of A and ρ. In addition, the target value A^* also depends on the choice of A and ρ. The task of HTA is to determine $\overline{\mathcal{A}} \in [c+\varepsilon_1, c-\varepsilon_1]$ or $\overline{\mathcal{A}} \notin [c+\varepsilon_2, c-\varepsilon_2]$ with $c = A^*$ for all inputs H in the form of shift-invariant and nearest-neighbor interaction, and thus to show undecidability we pick up a family of *bad* inputs depending on A, ρ, ε_1, and ε_2.

These two theorems are, in fact, different rewritings of the following technical lemma. This lemma claims that there exists a shift-invariant one-dimensional Hamiltonian which decodes the input code from the Hamiltonian itself and emulates the dynamics of a universal reversible Turing machine (URTM) properly. Note that in this lemma, $\boldsymbol{u}$ takes all possible binary bit strings: $\boldsymbol{u} \in \cup_{n=1}^{\infty}\{0,1\}^n$.

Lemma 1

Fix the dimension of the local Hilbert space at $d \geq d_0$. A complete orthonormal system (CONS) of the local Hilbert space $\{|e_i\rangle\}_{i=0}^{d-1}$ and an observable A over $\mathcal{H}$ with $\langle e_1|A|e_1\rangle = 0$ and $\langle e_2|A|e_2\rangle > 0$ are given arbitrarily. A universal reversible Turing machine (URTM) on a single tape is given arbitrarily. Then, for any $\eta > 0$ there exist a shift-invariant Hamiltonian H (which depends on the CONS) and a set of computable unitary operators $\{V_{\boldsymbol{u}}\}$ over $\mathcal{H}$ (which depends on the CONS, A, and $\eta > 0$) with the following properties:

- H consists of 1-body terms and 2-body nearest-neighbor interaction terms.
- For any $\boldsymbol{u}$, $V_{\boldsymbol{u}}|e_0\rangle = |e_0\rangle$ is satisfied.
- By setting the initial state as

$$|\psi_{V,L}\rangle := V_{\boldsymbol{u}}|e_0\rangle \otimes (V_{\boldsymbol{u}}|e_1\rangle)^{\otimes L-1}, \tag{S.8}$$

then with defining $\mathcal{V} := (V_{\boldsymbol{u}})^{\otimes L}$

$$\min\{\overline{\mathcal{A}}(H,\psi_V), \overline{\mathcal{V}\mathcal{A}\mathcal{V}^\dagger}(H,\psi_V)\} \geq \left(\frac{1}{4} - \eta\right)\langle e_2|A|e_2\rangle \tag{S.9}$$

is satisfied if and only if the URTM halts with the input $\boldsymbol{u}$, and

$$\max\{\overline{\mathcal{A}}(H,\psi_V), \overline{\mathcal{V}\mathcal{A}\mathcal{V}^\dagger}(H,\psi_V)\} \leq \eta \tag{S.10}$$

is satisfied if and only if the URTM does not halt with the input $\boldsymbol{u}$.

Here, we bound both $\overline{\mathcal{A}}$ and $\overline{\mathcal{V}\mathcal{A}\mathcal{V}^\dagger}$ in order to treat STA and HTA simultaneously. By this lemma, we can prove that the halting problem, a well-known undecidable problem, is not easier than STA nor HTA: So the latter is also undecidable.

We have discussed the undecidability of relaxation, where we consider whether the long-time average is close to a given value A^*. In the case of thermalization, we consider whether the long-time average is close to the equilibrium value $\mathrm{Tr}[\mathcal{A}\rho^{\mathrm{MC}}]$. Modifying slightly the proof techniques for Lemma 1, we can obtain HTA-type undecidability of thermalization. Here, we set $d_1 = d_0 + 5$.

Theorem 2

Fix the local dimension $d \geq d_1$. Let A be an arbitrary observable on a single site and ρ be an arbitrary pure state in the form of Eq. (S.7). We require that there exists states $|\phi_2\rangle$ and $|\phi_3\rangle$ orthogonal to each other and to $|\phi_0\rangle$, $|\phi_1\rangle$, $A|\phi_0\rangle$ and $A|\phi_1\rangle$ such that $\langle\phi_2|A|\phi_2\rangle > \max_{|\psi\rangle\in\mathrm{span}\{|\phi_0\rangle,|\phi_1\rangle\}} \langle\psi|A|\psi\rangle$ and $\langle\phi_3|A|\phi_3\rangle < \min_{|\psi\rangle\in\mathrm{span}\{|\phi_0\rangle,|\phi_1\rangle\}} \langle\psi|A|\psi\rangle$.
Then, for any large $M > 1$ there exist proper ε_2 and ε_1 with $\varepsilon_2 \geq \varepsilon_1 M$ such that the HTA for A and ρ with $c = \mathrm{Tr}[\mathcal{A}\rho^{\mathrm{MC}}]$, ε_2 and ε_1, is undecidable. This remains valid even if the Hamiltonian H (inputs of HTA) is restricted to a shift-invariant Hamiltonian H with 1-body terms and 2-body nearest-neighbor interaction terms.

The conditions of $|\phi_2\rangle$ and $|\phi_3\rangle$ mean that the initial state $|\phi_1\rangle$ and $|\phi_0\rangle$ is not at the edge of the spectrum of A. Except for such anomalous cases, almost all setups are expected to satisfy these conditions.

We clarify the dependency of quantities, which is similar to that of Theorem 1b: Both the observable A and the initial state ρ are arbitrary (as far as the condition for $|\phi_2\rangle$ and $|\phi_3\rangle$ is satisfied). Two parameters for the error, ε_1 and ε_2, depend on the choice of A and ρ. The task of HTA is to determine $\overline{\mathcal{A}} \in [c+\varepsilon_1, c-\varepsilon_1]$ or $\overline{\mathcal{A}} \notin [c+\varepsilon_2, c-\varepsilon_2]$ with $c = \mathrm{Tr}[\mathcal{A}\rho^{\mathrm{MC}}]$ for all inputs H in the form of shift-invariant and nearest-neighbor interaction, and thus to show undecidability we pick up a family of *bad* inputs depending on A, ρ, ε_1, and ε_2.

5 Proof of Theorem 1a and Theorem 1b (undecidability of relaxation) from Lemma 1

Below, without loss of generality, we suppose $\langle e_1|A|e_1\rangle = 0$, which can be met by subtracting a constant factor. We also set the target value c in STA and HTA to zero.

Proof of Theorem 1a. We fix the CONS $\{|e_i\rangle\}$ arbitrarily. For any given $\varepsilon > 0$ and A, we set the operator A' as

$$A' = \begin{cases} \varepsilon\,|e_2\rangle\langle e_2| + A & \text{if } \langle e_2|A|e_2\rangle \geq 0, \\ -\varepsilon\,|e_2\rangle\langle e_2| - A & \text{if } \langle e_2|A|e_2\rangle < 0. \end{cases} \tag{S.11}$$

This choice guarantees $\langle e_1|A'|e_1\rangle = 0$ and $|\langle e_2|A'|e_2\rangle| \geq \varepsilon$. We set the system Hamiltonian H as what is given in Lemma 1 with $\eta = \varepsilon/8M$.

With these H and η, we claim that the STA for the following state family for $\boldsymbol{u}$

$$\{|\psi_{V,L}\rangle = V|e_0\rangle \otimes (V|e_1\rangle)^{\otimes L-1} | V = V_{\boldsymbol{u}}\}_{\boldsymbol{u}} \tag{S.12}$$

with $\varepsilon_1 = \eta$ and $\varepsilon_2 = (1/4-\eta)\varepsilon$ is undecidable. The ratio of two errors is bounded as $\varepsilon_2/\varepsilon_1 = (1/4-\eta)\varepsilon \cdot 8M/\varepsilon \geq M$, where we used $\eta \leq 1/8$. The undecidability follows from Lemma 1, which suggests that

$$\overline{\mathcal{A}}(H, \psi_V) \geq \left(\frac{1}{4} - \eta\right)\langle e_2|A'|e_2\rangle \geq \left(\frac{1}{4} - \eta\right)\varepsilon = \varepsilon_2 \tag{S.13}$$

holds if and only if the URTM with the input $\boldsymbol{u}$ halts, and

$$\overline{\mathcal{A}}(H, \psi_V) \leq \eta = \varepsilon_1 \tag{S.14}$$

holds if and only if the URTM with the input $\boldsymbol{u}$ does not halt. Since the halting problem of the URTM is undecidable, this STA is also undecidable. □

Proof of Theorem 1b. We set a CONS $\{|e_i\rangle\}$ such that $|e_0\rangle$, $|e_1\rangle$, and $|e_2\rangle$ are the given states $|\phi_0\rangle$, $|\phi_1\rangle$, and $|\phi_2\rangle$, respectively, which ensures $\langle e_2|A|e_2\rangle \neq 0$. Without loss of generality, we can suppose $\langle e_2|A|e_2\rangle > 0$. (In case of $\langle e_2|A|e_2\rangle < 0$, we obtain an analogous conclusion by setting $A \to -A$.) Let H be the Hamiltonian whose existence is guaranteed by Lemma 1 with the above CONS, the above observable A, and $\eta = \langle e_2|A|e_2\rangle / 8M$. Using this H, we construct an A-dependent family of Hamiltonians for $\boldsymbol{u}$ given as

$$\{H_{V,L} = \mathcal{V}^\dagger H_L \mathcal{V} | \mathcal{V} = V_{\boldsymbol{u}}^{\otimes L}\}_{\boldsymbol{u}}. \tag{S.15}$$

We now set $\eta = \min(\langle e_2|A|e_2\rangle / 8M, 1/8)$ and $\varepsilon_1 = \eta$ and $\varepsilon_2 = (1/4 - \eta)\langle e_2|A|e_2\rangle$, which confirms $\varepsilon_2/\varepsilon_1 \geq (1/4 - \eta)\langle e_2|A|e_2\rangle \cdot 8M / \langle e_2|A|e_2\rangle \geq M$. By introducing a state family $|\psi_{V,L}\rangle = V_{\boldsymbol{u}}|e_0\rangle \otimes (V_{\boldsymbol{u}}|e_1\rangle)^{\otimes L-1}$, we have $\overline{\mathcal{A}}(H_V, \psi_I) = \overline{\mathcal{V}\mathcal{A}\mathcal{V}^\dagger}(H, \psi_V)$, and thus Lemma 1 implies that

$$\overline{\mathcal{A}}(H_V, \psi_I) = \overline{\mathcal{V}\mathcal{A}\mathcal{V}^\dagger}(H, \psi_V) \geq \left(\frac{1}{4} - \eta\right)\langle e_2|A|e_2\rangle = \varepsilon_2 \tag{S.16}$$

holds if and only if the URTM with the input $\boldsymbol{u}$ halts, and

$$\overline{\mathcal{A}}(H_V, \psi_I) = \overline{\mathcal{V}\mathcal{A}\mathcal{V}^\dagger}(H, \psi_V) \leq \eta = \varepsilon_1 \tag{S.17}$$

holds if and only if the URTM with the input $\boldsymbol{u}$ does not halt. Since the halting problem of the URTM is undecidable, the HTA with the initial state $|\psi_I\rangle = |\phi_0\rangle \otimes |\phi_1\rangle^{\otimes L-1}$, the observable A, ε_1 and ε_2 is also undecidable. □

6 Strategy for the proof of Lemma 1

Most of the remainder of this Supplementary Note, from Sec. 7 to Sec. 10, is devoted to proving Lemma 1. Before going to the details of the proof, we here sketch the strategy of the proof.

We first introduce a classical reversible Turing machine which our quantum system emulates (Sec. 7). As explained in Sec. 7.2, in order to implement it by a one-dimensional quantum system we generalize conventional Turing machines such that the finite control sits in the tape. The classical reversible Turing machine has two types of cells: M-cells and A-cells (see also Supplementary Figure 2). The decoding of an input code and simulation of a URTM is performed in M-cells, while A-cells are responsible for the change of $\overline{\mathcal{A}}$ (the long-time average of A) in case of halting. Namely, we emulate the URTM in M-cells, and if and only if the URTM halts, then we start flipping the state of A-cells. For a reason discussed later, we set the vast majority of cells to A-cells and a small fraction of cells to M-cells.

We construct a quantum system which emulates the above classical reversible Turing machine (Secs.8-10). The Hamiltonian is constructed in a similar manner to the Feynman-

Kitaev Hamiltonian without a clock: One-step time evolution of the classical Turing machine corresponds to an application of a quantum partial isometry as a quantum walk (Sec. 8). The idea of the Feynman-Kitaev Hamiltonian is first demonstrated with a simple toy example (Sec. 8.1), and then we construct the quantum isometry for our classical Turing machine (Sec. 8.2).

Since the dynamics of our system are a little complicated, we first introduce an analogous setting to our original one but easier to analyze, where the initial state is a single computational basis state representing the input code (Sec. 9). In this analogous setting, we demonstrate how the value of $\overline{\mathcal{A}}$ changes depending on the halting/non-halting of the URTM. In this case, the dynamics are fully solvable since the effective Hamiltonian for this initial state can be expressed as a tridiagonal matrix, which can be exactly diagonalized.

We then proceed to the original setting, a shift-invariant initial state (except site 1), where the initial state is a superposition of computational basis states (Sec. 10). The first two subsections (Sec. 10.1 and Sec. 10.2) are devoted to describing the decoding process of the input code for the URTM from an almost uniform initial state. With the help of the law of large numbers, we demonstrate that the input code is successfully decoded with arbitrarily high probability amplitude by choosing proper parameters. In the subsequent two subsections, we describe how the value of $\overline{\mathcal{A}}$ changes in the case of halting and non-halting. If the URTM halts, it starts flipping spins in A-cells to increase the value of A. The stable state of the corresponding quantum walk is uniformly distributed over all the states traversed by this discrete-time dynamics, so the expectation value of A becomes large if the URTM halts (Sec. 10.3). On the other hand, since the URTM is simulated on relatively small numbers of sites (M-cells), which are sparsely located in the 1D-chain, if the URTM does not halt, an overwhelming part of the system (A-cells) remains unchanged (Sec. 10.4).

7 Proof of Lemma 1: (1) Classical Turing machine

In this section, we construct a classical machine which is emulated by quantum many-body systems. Our classical machine consists of three TMs, *TM1*, *TM2*, and *TM3*, working on two types of cells, *M-cells* and *A-cells*. TM2 simulates a given URTM, and TM1 decodes its input from bit sequences. TM3 is responsible for changing the value of A when TM2 halts.

An M-cell has a three-layered structure: The first layer is a working space of TM1 and TM2, and the second and third layers, which take 0 or 1, contain the information of the input code for TM2. An A-cell has two distinct states; a_1 and a_2.

7.1 Universal reversible Turing machine

We fix a URTM characterized by the set $(Q, \Gamma, q_0, q_f, s_0, \delta)$. Here, Q is a set of states of the finite control, Γ is a set of tape alphabet symbols, q_0 and q_f are the initial and the final state of the finite control respectively, s_0 is the blank symbol, and δ is the transition function. The set Γ contains the symbol $\Box$, which stands for the leftmost cell. Without loss of generality, we suppose the followings:

1. We essentially use the quadruple form, that is, split each step into the two sub-steps: The first sub-step is rewriting the internal state and the symbol in the cell, and the second sub-step is the move of the tape head. To realize such moves of the machine, we set $Q := Q_m \times Q_u$ with $Q_m := \{m_0, m_1\}$, where $q_m \in Q_m$ is used to represent the

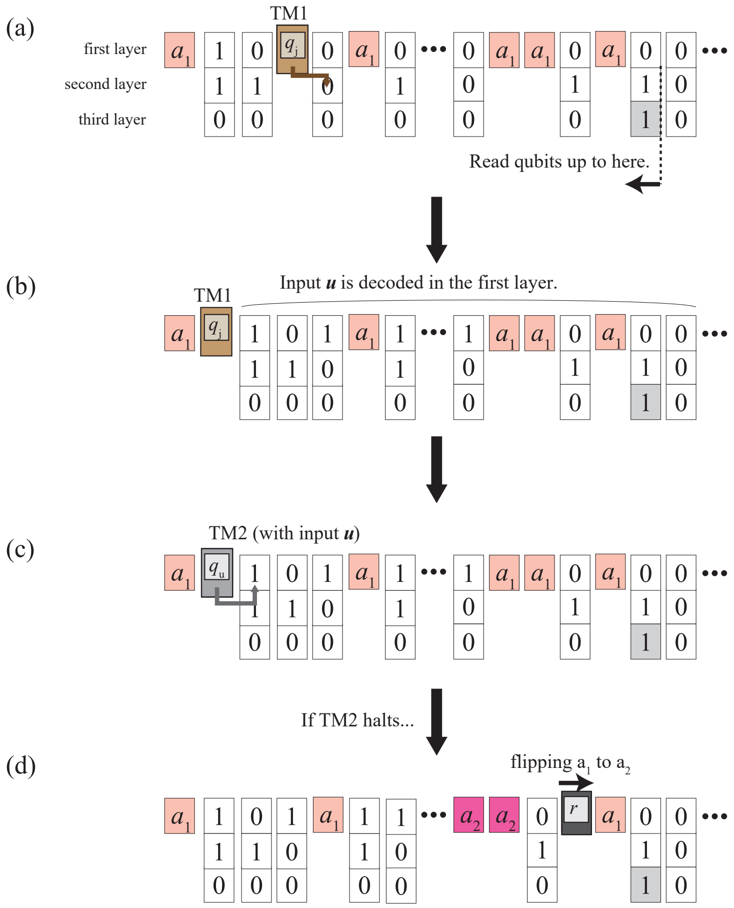

Supplementary Figure 2: Schematic of the dynamics and the structure of the emulated classical machine. Two types of cells, M-cell and A-cell, and a finite control sit in a single line. M-cells are three-layered colored in white, and A-cells are colored in red. In this figure, q_j, q_u, and r are the internal states of TM1, TM2, and TM3, respectively. (a) TM1 decodes the input code $\boldsymbol{u}$ for TM2 from the second and third layers of M-cells. The relative frequency of 1's in the second layer in the binary expansion is set to $\boldsymbol{u}$, and the leftmost 1 in the third layer (colored in gray) tells how long TM1 reads cells in the second layer and how many digits it decodes. Note that TM1 and TM2 pass through A-cells. (b) After decoding by TM1, the input code $\boldsymbol{u}$ is output to the first layer of M-cells. (c) When TM1 stops, then a universal reversible Turing machine TM2 starts working. If TM2 does not halt, TM2 eventually passes the periodic boundary, and then TM2 stops its move. (d) If TM2 halts, then TM3 start flipping the state in A-cells from a_1 to a_2, which changes the value of $\overline{\mathcal{A}}$.

two distinct sub-steps. It equals m_0 when rewriting and m_1 when moving the tape head. At every step, the value of q_m is flipped so that rewriting (other than the Q_m part) and moving on the tape occurs alternatingly. This is for the sake of clarity and at the same time to realize the movement of the tape head to the left by a two-body interaction.

2. We suppose that TM2 has *unique direction property* [10]: Q_u is split into three disjoint subsets, Q_+, Q_-, and Q_0 (i.e., $Q_u = Q_+ \cup Q_- \cup Q_0$). The state q_u is in Q_+ (resp. Q_-) if and only if the tape head has moved to the right (resp. left), and in Q_0 if and only if the tape head has not moved. We promise that $q_0 \in Q_m \otimes Q_+$ and $q_f \in Q_m \otimes Q_-$.

This property confirms the reversibility of this TM.

3. The tape head of the initial configuration is at the leftmost cell, and the Q_m part equals m_1. Hence, at the first step, it moves to the right.

7.2 Generalized URTM

To implement classical TMs by one-dimensional quantum many-body systems, we need some modifications to the above URTM. First, in conventional TMs the finite control sits outside the tape, while we will set the finite control in the tape. Second, we add two (non-universal) TMs, TM1 and TM3, which work before and after TM2 runs, respectively.

We first generalize the TM such that the finite control sits in the tape. Any RTM (reversible Turing machine) can be simulated by the following generalized URTM, as long as the length of the tape suffices. The generalized TM is almost the same as the URTM in the previous subsection, except for the following respects:

- The finite control sits between the two cells of the tape, and can read only one of the two adjacent cells in a single step. If it reads the cell right (left) to it, it moves to the right (left) or does not move. Each element q of the extended state space Q (we employ the same symbol for this extended space for brevity) tells which cell will interact with the finite control in this step.
- (Unique pairing property) For the sake of reversibility, we assume the following: Each element q of the state space Q tells the previous position of the finite control and the cell it has interacted with. In analogy with the unique direction property of an RTM, we call this property *a unique pairing property.*
- There are several configurations with no successor.

We next add TM1 and TM3. Since the detailed rule of TM1 will be presented in Sec. 10.1, we here briefly explain the dynamics of TM1 and TM3.

As explained, the second and third layers consist of 01-bit sequences. Let β be a real number whose binary expansion is equal to the input code $\boldsymbol{u}$, and set the relative frequency of 1 in the second layer to β. We also set the third layer such that almost all cells are filled with 0, and the state 1 rarely appears. TM1 counts the relative frequency of 1 in the second layer until we encounter the first 1 in the third layer.

If and only if TM2 halts, TM3 starts working. TM3 moves right, and if TM3 encounters an A-cell with the state a_1, it flips the state to a_2. If TM3 encounters an A-cell with the state a_2, which means that TM3 has moved around the system and goes back to the start point, then TM3 stops.

We call the composite classical machine of these TMs as generalized URTM and denote it by $M_{\rm G}$.

7.3 The states, symbols, and tape of $M_{\rm G}$

We define the set of states and symbols of $M_{\rm G}$ as

$$Q \cup Q_r = (Q_m \times Q_u) \cup Q_r, \tag{S.18}$$

$$\overline{\Gamma} \times \Gamma_{\rm in} \times \Gamma_A = (\Gamma \cup \{s_1\}) \times (\{0,1\}^{\otimes 2} \cup \{\psi_0\}) \times \Gamma_A, \tag{S.19}$$

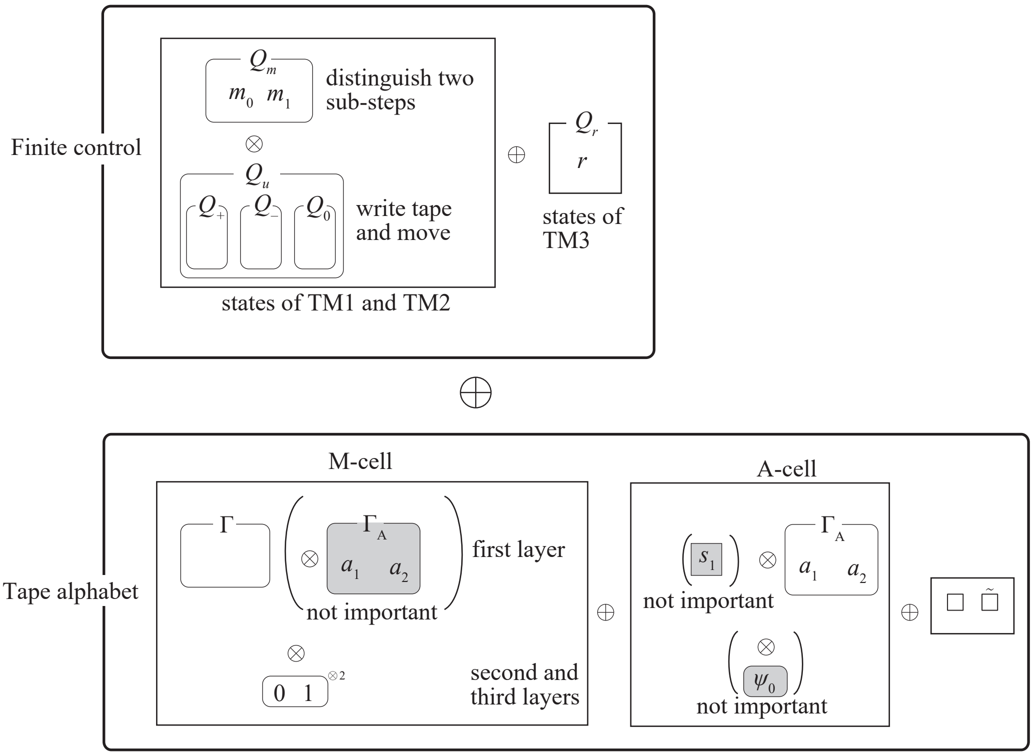

Supplementary Figure 3: Summary of possible states of a single site in the quantum system. The symbols in gray areas are dummy states which are not important.

where we set

$$Q_m := \{m_0, m_1\}, \tag{S.20}$$

$$Q_r := \{r\}, \tag{S.21}$$

$$\overline{\Gamma} := \Gamma \cup \{s_1\}, \tag{S.22}$$

$$\Gamma_A := \{a_1, a_2\}. \tag{S.23}$$

Here, $Q_m \times Q_u$ represents the states of the finite control of TM1 and TM2, and $Q_r = \{r\}$ is the sign for TM3, which flips the states in A-cells. The alphabets Γ, Γ_{in} and Γ_A appear in the first layer of M-cells, the second and third layers of M-cells, and A-cells, respectively. The symbol s_1 and ψ_0 are signs that this cell is an A-cell. Note that Γ contains the blank symbol s_0. We compile these symbols in Supplementary Figure 3.

Out of the $L-1$ cells, $\lfloor \alpha(L-1) \rfloor$ cells are filled with symbols from $\Gamma \times \Gamma_A$, which serve as M-cells, and $\lceil (1-\alpha)(L-1) \rceil$ cells are filled with $\{s_1\} \times \Gamma_A$, which serve as A-cells. M-cells simulate TM1 and TM2, and A-cells inflate the value of the observable A in case of halting. The rate α will be taken sufficiently small to observe the gap between the halting case and the non-halting case (i.e., almost all cells are set as A-cells, though the number of M-cells is also sufficiently large in $L \to \infty$ limit).

Initially, M_{G}'s state is set to (m_0, q_0), and the Γ_A-part of the cell is set to a_1. Cells in the left of the finite control are set to the blank cell s_0. Below, for brevity we leave off the symbols $\lfloor \cdot \rfloor$ and $\lceil \cdot \rceil$ and also drop -1, as L is large enough (i.e., we write αL and $(1-\alpha)L$

instead of $\lfloor \alpha(L-1) \rfloor$ and $\lceil (1-\alpha)(L-1) \rceil$).

7.4 The move of M_{G}: the first step

When M_{G} is in the initial state (m_0, q_0), this RTM not only mimics the move of TM1 but also prepares the cell with $\Box$. The finite control reads the site on its right and rewrites the cell as $s_0 \to \Box$ and $s_1 \to \Box'$ (There is no other possibility in legal configurations). We employ two different symbols $\Box$ and $\Box'$ only for satisfying the reversibility, and $\Box$ and $\Box'$ plays completely the same role: telling the left/right end of the tape. Both TM1 and TM2 stop if they hit these cells, while TM3 just passes this cell transparently. Therefore, in the remainder of this Supplementary Note, we do not distinguish $\Box'$ from $\Box$.

At the first step, the state and the position of the finite control will be changed in the same manner as the TM1's first step. Hence, first, it evolves to (m_1, q), and then it moves to the right and evolves to (m_0, q).

7.5 The move of M_{G}: simulating TM1 and TM2

While the state of the finite control q is in Q, M_{G} simulates TM1 and TM2 in M-cells, and it virtually ignores A-cells (see Supplementary Figure 2.(a)-(c)).

- If $q_m = m_0$, it reads the cell in its right. If it is an M-cell, the machine updates its state of Q_u part, q_u say, and the symbol in the cell according to the transition function δ. At the same time, Q_m part of the finite control, q_m say, evolves to m_1. If it is an A-cell, q_u does not change and q_m simply evolves to m_1.
- If $q_m = m_1$ and $q_u \in Q_+$, the finite control is swapped with the cell in its right, and q_m evolves to m_0.
- If $q_m = m_1$ and $q_u \in Q_-$, the finite control is swapped with the cell in its left, and q_m evolves to m_0.
- If $q_m = m_1$ and $q_u \in Q_0$, the finite control does not move, and q_m simply evolves to m_0.

If $q_m = m_1$, the previous partner of the finite control is the cell on its right, and its previous position is the same as the present position. If $q_m = m_0$ and $q_u \in Q_+$ (resp. $q_u \in Q_-$), the previous partner of the finite control is the cell on the left (resp. right) of it, and the previous position is at the left (resp. right) of the previous partner.

Eventually, the machine may run out of the tape: In case of the periodic boundary condition, the finite control reads the symbol $\Box$ with $q_u \in Q_+ \backslash \{q_0\}$. Since these states are illegal configurations, no successor is defined to them.

If the machine runs into the halting state q_f, then the finite control evolves into the state $r \in Q_r$. The dynamics after halting are given in the next subsection.

7.6 The move of M_{G}: simulating TM3

If TM2 halts and the state of the finite control becomes in $q \in Q_r$ (i.e., $q = r$), then TM3 starts flipping the state in A-cells from a_1 to a_2 (Supplementary Figure 2.(d)). If the cell on the right of the finite control is an A-cell with its state a_1, then the head flips the state to a_2 and moves right. If the cell on the right of the finite control is an M-cell, then the finite

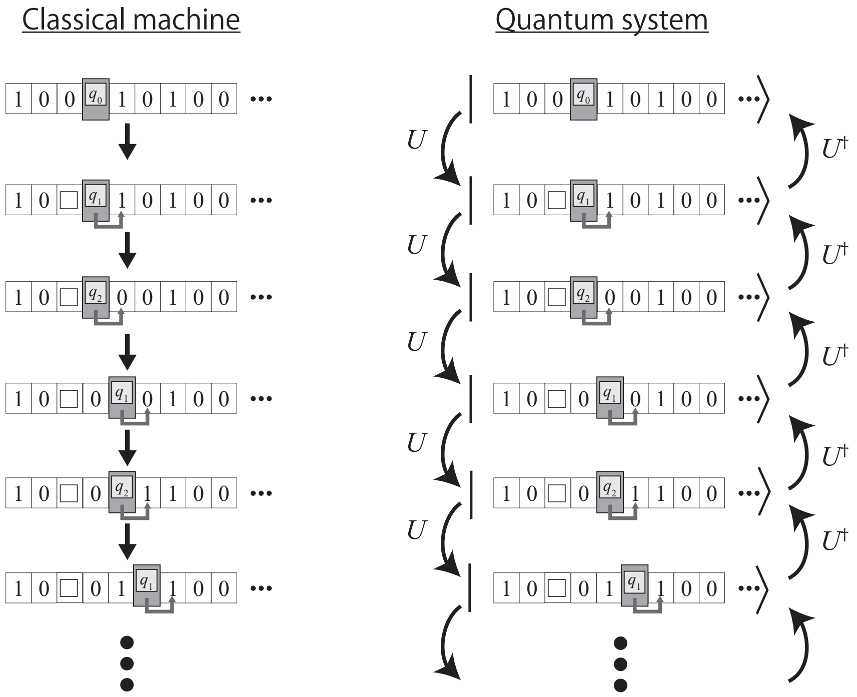

Supplementary Figure 4: The dynamics of the toy example of a classical machine introduced in Sec. 8.1, and the corresponding quantum partial isometry U.

control just moves right. If the cell on the right of the finite control is an A-cell with its state a_2, which implies that all of the A-cells have already been flipped to a_2 and the finite control has gone around the periodic system, then TM3 stops.

8 Proof of Lemma 1: (2) Quantum partial isometry corresponding to M_{G}

8.1 Toy example of Feynman-Kitaev type Hamiltonian

Before constructing the quantum partial isometry for M_{G}, for readers who are not familiar with the emulation of classical machines by quantum systems, we here demonstrate a toy example of a quantum partial isometry emulating a very simple classical machine. If a reader is familiar with this topic, one can skip this subsection and start reading the subsection entitled *Construction of quantum partial isometry for* M_{G}.

We consider a simple classical machine, flipping bits with moving from left to right on a single cell line. The finite control also settles in this line. The finite control takes three possible states, q_0, q_1 and q_2, and the transition rule of this machine is the following (see also Supplementary Figure 4):

1. If the state of the finite control is q_0, then the state of the finite control evolves to q_1. At the same time, the cell on the left of the finite control is flipped to $\square$ (if the cell is 0) or $\tilde{\square}$ (if the cell is 1).

2. If the state of the finite control is q_1 and the cell on the right of the finite control is neither $\Box$ nor $\tilde{\Box}$, then the machine flips the bit (i.e., $0 \to 1$ and $1 \to 0$) in the cell on the right of the finite control. At the same time, the state of the finite control evolves to q_2.

3. If the state of the finite control is q_2, then the finite control moves to the right. At the same time, the state of the finite control evolves to q_1.

4. If the state of the finite control is q_1 and the cell on the right of the finite control is $\Box$ or $\tilde{\Box}$, then the machine stops.

We emulate the above classical machine by a one-dimensional quantum system with nearest-neighbor interaction. The local Hilbert space is spanned by the following seven states:

$$\{|0\rangle, |1\rangle, |q_0\rangle, |q_1\rangle, |q_2\rangle, |\Box\rangle, |\tilde{\Box}\rangle\}.$$

The local quantum isometry on the sites i and $i+1$ is given as

$$U_{i,i+1} = U^1_{i,i+1} + U^2_{i,i+1} + U^3_{i,i+1} \tag{S.24}$$

with

$$U^1 = |\Box q_1\rangle \langle 0 q_0| + |\tilde{\Box} q_1\rangle \langle 1 q_0|, \tag{S.25}$$
$$U^2 = |q_2 1\rangle \langle q_1 0| + |q_2 0\rangle \langle q_1 1|, \tag{S.26}$$
$$U^3 = |1 q_1\rangle \langle q_2 1| + |0 q_1\rangle \langle q_2 0|, \tag{S.27}$$

which correspond to the transition rules 1,2,3, respectively. The quantum isometry of the total system is given by

$$U = \sum_i U_{i,i+1}. \tag{S.28}$$

The initial state is restricted to the form that only a single site takes $|q_0\rangle$ and other sites are in the local Hilbert subspace spanned by $\{|0\rangle, |1\rangle\}$, which ensures the emulation of legal states of the classical machine. By denoting by $|\psi_n\rangle$ the state representing the state of the classical machine at the n-th step, then

$$U |\psi_n\rangle = |\psi_{n+1}\rangle \tag{S.29}$$

is satisfied, which means that the quantum isometry U induces one step evolution of the quantum state.

8.2 Construction of quantum partial isometry for M_{G}

Now we consider our original Turing machine. By defining

$$X := Q \cup Q_r \cup \{\overline{\Gamma} \times \Gamma_A\}, \tag{S.30}$$

then the sequence of the elements $\boldsymbol{x} = (x_1, x_2, \ldots, x_L)$ $(x_i \in X)$ represents an instantaneous configuration of M_{G}, where only one of x_i's is a member of $Q \cup Q_r$. We here dropped the second and third layers of M-cells (i.e., Γ_{in} from $\{\overline{\Gamma} \times \Gamma_A\}$) for brevity, which are explicitly

treated in Sec. 10.1. The last condition on $\boldsymbol{x}$ ensures a single finite control and a single tape head in the system.

We denote by $\mathcal{H}^X$ the Hilbert space spanned by $\{|x\rangle \,|x \in X\}$, and define $\mathcal{H}^Q$, $\mathcal{H}^{Q_m}$, $\mathcal{H}^{Q_u}$, $\mathcal{H}^{Q_r}$, $\mathcal{H}^{\overline{\Gamma}}$ and $\mathcal{H}^{\Gamma_A}$ in a similar manner, which satisfy

$$\mathcal{H}^X = \mathcal{H}^Q \oplus \mathcal{H}^{Q_r} \oplus (\mathcal{H}^{\overline{\Gamma}} \otimes \mathcal{H}^{\Gamma_A}), \tag{S.31}$$

$$\mathcal{H}^Q = \mathcal{H}^{Q_m} \otimes \mathcal{H}^{Q_u}. \tag{S.32}$$

A quantum state $|\boldsymbol{x}\rangle \in (\mathcal{H}^X)^{\otimes L}$ represents $\boldsymbol{x}$, a configuration of M_{G}. Since the states in $\mathcal{H}^X$ are only the label (except for Γ_A), we assign the CONS given in Lemma 1 to the CONS $\{|x\rangle \,|x \in X\}$ as

$$|m_0, q_0\rangle = |e_0\rangle\,, \tag{S.33}$$

$$|s_1, a_i\rangle = |e_i\rangle\,, \quad (i = 1, 2). \tag{S.34}$$

With this assignment, the value of A indeed varies between $i = 1$ and $i = 2$ with the state s_1:

$$\langle s_1, a_1|A|s_1, a_1\rangle = \langle e_1|A|e_1\rangle = 0, \tag{S.35}$$

$$\langle s_1, a_2|A|s_1, a_2\rangle = \langle e_2|A|e_2\rangle > 0. \tag{S.36}$$

Each step of the move (time evolution) of M_{G} can be described by a partial isometry acting on at most two sites simultaneously. Thus, the sum of the partial isometries acting on at most two sites properly emulates the time evolution of M_{G}. Suppose that in M_{G} the tape head is at the i-th cell, or equivalently $x_i \in Q \cup Q_r$. We set the partial isometry U_i acting on the $i-1$, i, and $i+1$-th sites as

$$U_i = U_i^0 + U_i^{1+} + U_i^{1-} + U_i^{10} + U_i^r, \tag{S.37}$$

where each summand acts on at most two sites. If the state of the finite control is in (m_0, q), the move of the machine is emulated[9] by U_i^0. If the i-th and $i+1$-th cells in M_{G} evolve from (x_i, x_{i+1}) to (x_i', x_{i+1}'), the corresponding isometry on the sites i and $i+1$ is given by $|x_i', x_{i+1}'\rangle \langle x_i, x_{i+1}|$. If the state of the finite control is in (m_1, q), the move is emulated by one of U_i^{1+}, U_i^{1-}, or U_i^{10}, depending on whether $q \in Q_+$, Q_-, or Q_0. They swap the sites and change m_1 to m_0. U_i^r mimics the move of TM3 if the state is $r \in Q_r$ and the finite control is at i-th site.

We note that if the i-th site does not correspond to the finite control (i.e., $x_i \notin Q \cup Q_r$), then $U_i\,|\boldsymbol{x}\rangle = 0$, which ensures the fact that only the vicinity of the finite control can change. Owing to this, the isometry on $(\mathcal{H}^X)^{\otimes L}$ given by

$$U = \sum_i U_i \tag{S.38}$$

properly emulates the dynamics of the generalized RTM M_{G}.

[9] This is represented by an isometry from $\mathbb{C}(|m_0\rangle \otimes \mathcal{H}^{Q_u})_i \otimes (\mathcal{H}^{\overline{\Gamma}} \otimes \mathcal{H}^{\Gamma_A})_{i+1}$ to the same Hilbert space.

9 Proof of Lemma 1: (3) Evaluating $\overline{\mathcal{A}}$ for computational basis state

Since the computation of $\overline{\mathcal{A}}$ from the almost uniform initial state $|\phi_0\rangle \otimes |\phi_1\rangle^{\otimes L-1}$ is a little complicated, in this section we first consider an easier setting with a computational basis initial state. With this initial state, We treat almost uniform initial states in the next section.

9.1 General expression of long-time average

We first derive a general expression of the long-time average $\overline{\mathcal{A}}$. Let H be a Hamiltonian of the system, and E_i and $|E_i\rangle$ be eigenenergy and corresponding energy eigenstate. We expand the initial state $|\psi\rangle$ with the energy eigenbasis as $|\psi\rangle = \sum_i c_i |E_i\rangle$. Then, the long-time average of an observable A is calculated as

$$\begin{aligned}\overline{\mathcal{A}} &= \lim_{T\to\infty} \frac{1}{T}\int_0^T dt \, \langle \psi(t)|A|\psi(t)\rangle \\ &= \lim_{T\to\infty} \frac{1}{T}\int_0^T dt \sum_{i,j} e^{-i(E_j-E_i)t} c_i^* c_j \langle E_i|A|E_j\rangle \\ &= \sum_{i,j} \chi(E_j = E_i) c_i^* c_j \langle E_i|A|E_j\rangle \, , \end{aligned} \tag{S.39}$$

where $\chi(E_j = E_i)$ takes 1 if $E_j = E_i$ and takes zero otherwise. We set the Planck constant to unity. In the third line, we used the fact that $\lim_{T\to\infty} \frac{1}{T}\int_0^T dt e^{-i(E_j-E_i)t}$ converges to zero if $E_j \neq E_i$ and equal to 1 if $E_j = E_i$.

In particular, if the Hamiltonian H has no degeneracy, we have a simple expression $\overline{\mathcal{A}} = \sum_i |c_i|^2 \langle E_i|A|E_i\rangle$. In contract, if there exist degeneracy ($E_i = E_j$ with $i \neq j$), we need to handle off-diagonal elements $\langle E_1|A|E_j\rangle$.

9.2 Hamiltonian emulating M_{G} and effective Hamiltonian

We now construct the Hamiltonian of the quantum system emulating M_{G}. We define the Hamiltonian as

$$H = \sum_i (U_i + U_i^\dagger), \tag{S.40}$$

where U_i is introduced in Eq. (S.37).

Let $\boldsymbol{x}^j$ be the configuration of M_{G} at the j-th step, and J be the number of time steps until the machine stops. Then, its corresponding quantum state $|\boldsymbol{x}^j\rangle$ satisfies

$$|\boldsymbol{x}^j\rangle = U^{j-1} |\boldsymbol{x}^1\rangle \, , \tag{S.41}$$

and our Hamiltonian restricted to the Hilbert space spanned by $\{\boldsymbol{x}^i\}_{i=1}^J$ can be expressed as

$$H_{\mathrm{eff}} := \sum_{j=1}^{J-1} |\boldsymbol{x}^{j+1}\rangle \langle \boldsymbol{x}^j| + \mathrm{h.c.}. \tag{S.42}$$

Note that $\boldsymbol{x}^J$ does not have a successor, and hence the evolution of M_{G} stops at this point.

Since $H_{\rm eff}$ is a tridiagonal matrix with the basis $\{|\boldsymbol{x}^i\rangle\}_{i=1}^J$, we can fully solve its eigenenergies and eigenstates. The k-th eigenenergy is written as

$$E_k = 2\cos\left(\frac{k\pi}{J+1}\right) \tag{S.43}$$

($k = 1, 2, \dots, J$) with the corresponding energy eigenstate

$$|E_k\rangle = \sqrt{\frac{2}{J+1}}\sum_{j=1}^{J}\sin\left(\frac{kj\pi}{J+1}\right)|\boldsymbol{x}^j\rangle\,. \tag{S.44}$$

Note that $0 < \frac{k\pi}{J+1} < \pi$ guarantees the absence of degeneracy in this Hamiltonian.

9.3 Computing long-time average of $\mathcal{A}$

The initial state $|\boldsymbol{x}^1\rangle$ is expanded by the energy eigenstates as

$$|\boldsymbol{x}^1\rangle = \sqrt{\frac{2}{J+1}}\sum_{k=1}^{J}\sin\left(\frac{k\pi}{J+1}\right)|E_k\rangle\,, \tag{S.45}$$

and thus $\overline{\mathcal{A}}$ is calculated as

$$\begin{aligned}
\overline{\mathcal{A}} &= \frac{2}{J+1}\sum_{k=1}^{J}\sin^2\left(\frac{k\pi}{J+1}\right)\langle E_k|\mathcal{A}_L|E_k\rangle \\
&= \left(\frac{2}{J+1}\right)^2\sum_{j,j'=1}^{J}\left[\sum_{k=1}^{J}\sin^2\left(\frac{k\pi}{J+1}\right)\sin\frac{j'k\pi}{J+1}\sin\frac{jk\pi}{J+1}\right]\langle\boldsymbol{x}^j|\mathcal{A}_L|\boldsymbol{x}^{j'}\rangle \\
&= \frac{3}{2(J+1)}\left(\langle\boldsymbol{x}^1|\mathcal{A}_L|\boldsymbol{x}^1\rangle + \langle\boldsymbol{x}^J|\mathcal{A}_L|\boldsymbol{x}^J\rangle\right) + \frac{1}{J+1}\sum_{j=2}^{J-1}\langle\boldsymbol{x}^j|\mathcal{A}_L|\boldsymbol{x}^j\rangle - \frac{1}{2(J+1)}\sum_{\substack{1\le j,j'\le J\\ j=j'\pm 2}}\langle\boldsymbol{x}^j|\mathcal{A}_L|\boldsymbol{x}^{j'}\rangle\,.
\end{aligned} \tag{S.46}$$

Here, in the first line we used Eq. (S.39), and in the last line we used the following relation:

$$\sum_{k=1}^{J}\sin^2\left(\frac{k\pi}{J+1}\right)\sin\frac{j'k\pi}{J+1}\sin\frac{jk\pi}{J+1} = \begin{cases}\frac{1}{4}(J+1) & j=j'\neq 1 \text{ and } \neq J,\\ \frac{3}{8}(J+1) & j=j'=1 \text{ or } J,\\ -\frac{1}{8}J(J+1) & j=j'\pm 2,\\ 0 & j\neq j' \text{ and } j\neq j'\pm 2.\end{cases} \tag{S.47}$$

Now, we shall show that the last term $\langle\boldsymbol{x}^j|\mathcal{A}_L|\boldsymbol{x}^{j'}\rangle$ is sufficiently small. Since $\boldsymbol{x}^j\neq\boldsymbol{x}^{j'}$ and $\mathcal{A}_L$ is a sum of one-body observables; $\mathcal{A}_L = \frac{1}{L}\sum_i A_i$, we find that $\langle\boldsymbol{x}^j|\mathcal{A}_L|\boldsymbol{x}^{j'}\rangle$ can take a nonzero value only if $\boldsymbol{x}^j$ and $\boldsymbol{x}^{j'}$ differs only in a single site. By denoting this site by i^*, we bound the last term $\langle\boldsymbol{x}^j|\mathcal{A}_L|\boldsymbol{x}^{j'}\rangle$ as

$$\left|\langle\boldsymbol{x}^j|\mathcal{A}_L|\boldsymbol{x}^{j'}\rangle\right| = \frac{1}{L}\left|\sum_{i=1}^{L}\langle\boldsymbol{x}^j|A_i|\boldsymbol{x}^{j'}\rangle\right| = \frac{1}{L}\left|\langle\boldsymbol{x}^j|A_{i^*}|\boldsymbol{x}^{j'}\rangle\right| \le \frac{1}{L}\|A\|. \tag{S.48}$$

Thus, the last term of Eq. (S.46) is bounded above as

$$\left| \frac{1}{2(J+1)} \sum_{\substack{1\le j,j'\le J \\ j=j'\pm 2}} \langle \boldsymbol{x}^j | \mathcal{A}_L | \boldsymbol{x}^{j'} \rangle \right| \le \left| \frac{1}{2(J+1)} \sum_{\substack{1\le j,j'\le J \\ j=j'\pm 2}} \frac{1}{L} \|A\| \right| = \frac{J-2}{J+1} \frac{1}{L} \|A\|, \tag{S.49}$$

whose right-hand side vanishes in the $L \to \infty$ limit. Therefore, in the following, we dropped this term for brevity.

We finally evaluate the first two terms of Eq. (S.46). Let $N_A(\boldsymbol{x}^j)$ be the number of A-cells in $\boldsymbol{x}^j$ filled with a_2. Then, we have

$$\left| \langle \boldsymbol{x}^j | \mathcal{A}_L | \boldsymbol{x}^j \rangle - \frac{N_A(\boldsymbol{x}^j)}{L} \langle e_2 | A | e_2 \rangle \right| \le \alpha \|A\|, \tag{S.50}$$

where the difference between $\langle \boldsymbol{x}^j | \mathcal{A}_L | \boldsymbol{x}^j \rangle$ and $\frac{N_A(\boldsymbol{x}^j)}{L} \langle e_2 | A | e_2 \rangle$ comes from the presence of M-cells. In the case of halting, by taking L sufficiently large, we can make M_{G} halt before $J/2$ steps. In this condition, $\frac{N_A(\boldsymbol{x}^j)}{L} \ge 2(j - \frac{J}{2})$ is satisfied for $j \ge \frac{J}{2}$, which indicates

$$\overline{\mathcal{A}} = \frac{3}{2(J+1)} \left(\langle \boldsymbol{x}^1 | \mathcal{A}_L | \boldsymbol{x}^1 \rangle + \langle \boldsymbol{x}^J | \mathcal{A}_L | \boldsymbol{x}^J \rangle \right) + \frac{1}{J+1} \sum_{j=2}^{J-1} \langle \boldsymbol{x}^j | \mathcal{A}_L | \boldsymbol{x}^j \rangle \ge \frac{1}{4} \langle e_2 | A | e_2 \rangle - \alpha \|A\|. \tag{S.51}$$

By taking α sufficiently small, we arrive at the relation $\overline{\mathcal{A}} \ge \left(\frac{1}{4} - \eta \right) \langle e_2 | A | e_2 \rangle$ in case of halting.

In contrast, in case of non-halting, since $N_A(\boldsymbol{x}^j) = 0$ for any j, we have

$$\overline{\mathcal{A}} = \frac{3}{2(J+1)} \left(\langle \boldsymbol{x}^1 | \mathcal{A}_L | \boldsymbol{x}^1 \rangle + \langle \boldsymbol{x}^J | \mathcal{A}_L | \boldsymbol{x}^J \rangle \right) + \frac{1}{J+1} \sum_{j=2}^{J-1} \langle \boldsymbol{x}^j | \mathcal{A}_L | \boldsymbol{x}^j \rangle \le \alpha \|A\|. \tag{S.52}$$

By taking α sufficiently small, we arrive at the relation $\overline{\mathcal{A}} \le \eta$ in case of non-halting.

10 Proof of Lemma 1: (4) Evaluating $\overline{\mathcal{A}}$ for superposition of computational basis states

10.1 Setting of the initial state and decoding of the input

We now consider our original setting where the initial state is shift-invariant except the first site, which takes the form of

$$|\psi_{V,L}\rangle = (V |e_0\rangle) \otimes (V |e_1\rangle)^{\otimes L-1} = |e_0\rangle \otimes (V |e_1\rangle)^{\otimes L-1}. \tag{S.53}$$

Here, $|e_0\rangle$ represents the state corresponding to the initial state of the finite control, and others represent cells.

As announced in Sec. 8.2, we first elongate the local Hilbert subspace to $\mathcal{H}^{\Gamma} \otimes \mathcal{H}^{\Gamma_A} \otimes \mathcal{H}^{\mathrm{in}}$ in order to treat the alphabets in Γ_{in} in M_{G}, which represent the second and third layers of M-cells. The Hilbert space $\mathcal{H}^{\mathrm{in}}$ is a 5-dimensional space which is a sum of a 2 qubit space

and a single state space $\{|\psi_0\rangle\}$:

$$\mathcal{H}^{\text{in}} = \text{span}\{\{|0\rangle, |1\rangle\}^{\otimes 2} \oplus |\psi_0\rangle\}. \tag{S.54}$$

The input code for TM2 is encoded into $|\psi_{\text{in}}\rangle \in \{|0\rangle, |1\rangle\}^{\otimes 2} \subset \mathcal{H}^{\text{in}}$, which sits in all M-cells. If the cell is an A-cell, this part is blank (constant independent of the input) denoted by $|\psi_0\rangle \in \mathcal{H}^{\text{in}}$.

In the previous section, we set αL cells to M-cells and $(1-\alpha)L$ cells to A-cells deterministically. In this section, instead of this, we set $V|e_1\rangle$ as a superposition of these two types of cells. We first assign $|e_1\rangle$ and $|e_2\rangle$ as

$$|s_1, a_k\rangle |\psi_0\rangle = |e_k\rangle \in \mathcal{H}^{\overline{\Gamma}} \otimes \mathcal{H}^{\Gamma_A} \otimes \mathcal{H}^{\text{in}}, \quad (k = 1, 2), \tag{S.55}$$

and then apply an operator V which slightly rotates $|e_k\rangle$ $(k = 1, 2)$ as

$$V|e_k\rangle = \sqrt{\alpha}\, |s_0, a_k\rangle |\psi_{\text{in}}\rangle + \sqrt{1-\alpha}\, |e_k\rangle. \tag{S.56}$$

Here, two states, $|s_0, a_1\rangle |\psi_{\text{in}}\rangle$ and $|e_1\rangle = |s_1, a_1\rangle |\psi_0\rangle$, correspond to the initial states of M-cells and A-cells, respectively (see also Supplementary Figure 3). The symbol s_0 serves as a blank cell of M-cells in the first layer, and $|\psi_{\text{in}}\rangle \in \mathcal{H}^{\text{in}}$ stores the input $\boldsymbol{u}$ for TM2 in the second and third layers of M-cells. In the state $|s_0, a_k\rangle |\psi_{\text{in}}\rangle$, the symbol a_k plays no role. In the state $|s_1, a_k\rangle |\psi_0\rangle$, the symbol s_1 is a sign to be A-cells, and $|\psi_0\rangle$ plays no role. The symbols a_1 and a_2 distinguish two states of A-cells, which change the value of A. We set the operator V as an identity operator on the orthogonal component of $\mathcal{H}^{\overline{\Gamma}} \otimes \mathcal{H}^{\Gamma_A} \otimes \mathcal{H}^{\text{in}}$. In particular, V stabilizes $|e_0\rangle$ (i.e., $V|e_0\rangle = |e_0\rangle$).

The state $|\psi_{\text{in}}\rangle$ takes the form of

$$|\psi_{\text{in}}\rangle = (\sqrt{\beta}\, |1\rangle + \sqrt{1-\beta}\, |0\rangle) \otimes (\sqrt{\gamma}\, |1\rangle + \sqrt{1-\gamma}\, |0\rangle), \tag{S.57}$$

where the binary expansion of β is set to be equal to the input code $\boldsymbol{u}$ in the form of a binary bit string. The amount of β is guessed by the relative frequency of 1's in the second layer (see also Supplementary Figure 2.(a)). The second layer is a superposition of computational basis states, and thus TM1 runs in each computational basis state as a quantum superposition. Consider m copies of $\sqrt{\beta}\, |1\rangle + \sqrt{1-\beta}\, |0\rangle$, which is expanded as

$$(\sqrt{\beta}\, |1\rangle + \sqrt{1-\beta}\, |0\rangle)^{\otimes m} = \sum_{\boldsymbol{w} \in \{0,1\}^{\otimes m}} \sqrt{\beta}^{N_1(\boldsymbol{w})} \sqrt{1-\beta}^{m-N_1(\boldsymbol{w})} |\boldsymbol{w}\rangle. \tag{S.58}$$

Here, $N_1(\boldsymbol{w})$ is the number of 1's in the binary sequence $\boldsymbol{w}$. The probability amplitude for a computational basis state $|\boldsymbol{w}\rangle$ is $|c_{\boldsymbol{w}}|^2 = \beta^{N_1(\boldsymbol{w})}(1-\beta)^{m-N_1(\boldsymbol{w})}$. Due to the law of large numbers, the probability amplitude for states where the relative frequency of 1's is close to β converges to 1 in the large m limit:

$$\lim_{m\to\infty} \sum_{\boldsymbol{w}:\, \frac{N_1(\boldsymbol{w})}{m} \simeq \beta} |c_{\boldsymbol{w}}|^2 = 1, \tag{S.59}$$

where the precise meaning of the symbol $\frac{N_1(\boldsymbol{w})}{m} \simeq \beta$ is clarified shortly (in Eq. (S.61)). Hence, if m is sufficiently large compared to the length of the input code, TM1 guesses β

correctly from the frequency of 1's.

The length of qubit m is determined by another bit sequence $\sqrt{\gamma}\,|1\rangle + \sqrt{1-\gamma}\,|0\rangle$ in the third layer. For any given accuracy $0 < \xi < 1$, the information of m is encoded to γ as satisfying

$$(1-\gamma)^m \geq 1-\xi. \tag{S.60}$$

As γ set to extremely close to 0, almost all qubits are $|0\rangle$ in this sequence, and the state $|1\rangle$ rarely appears. In particular, $|1\rangle$ appears only after m-th digit with probability larger than $1-\xi$. Owing to this, if $|1\rangle$ appears at the m'-th digit for the first time, this is taken as the sign of $m \leq m'$. Based on the observed value m', the length of the output by TM1 (i.e., the presumed length of the digit of β) is determined as $n' = \lceil \frac{1}{4}\log_2 m' \rceil$, which ensures

$$\lim_{m'\to\infty} \mathrm{Prob}\left[\left|\frac{N_1(\boldsymbol{w})}{m'} - \beta\right| < \frac{1}{2^{n'+1}}\right] = 1. \tag{S.61}$$

With this choice of the output length n', guessing m larger than the true value does not affect the correctness of the estimation of β.

10.2 Case when TM2 halts 1: decoding and expression of states

Suppose that TM2 halts on the encoded input. We claim that in this case, with overwhelming probability amplitude the dynamics by U before TM2 halts are described by the configuration of the first L_0 of M-cells, where L_0 is sufficiently large in the following sense:

- It is not smaller than the space used by TM1 and TM2.
- It should be large enough to encounter at least a single $|1\rangle$ in the third layer in the first L_0 of M-cells with high probability. In other words, for a given $0 < \xi, \xi' < 1$ we set γ and L_0 such that

$$\begin{aligned}(1-\gamma)^m &\geq 1-\xi,\\ (1-\gamma)^{L_0} &\leq 1-\xi',\end{aligned} \tag{S.62}$$

  where the first line is the same as Eq. (S.60). By setting $\xi, \xi' \ll 1$, the first $|1\rangle$ in the second layer appears between the first m of M-cells and the first L_0 of M-cells with high probability.

We emphasize that L_0 is independent of the system size L.

Let C be the cluster of the first $|C|$ sites with

$$|C| = \frac{L_0}{\alpha} + o(L_0). \tag{S.63}$$

Here, the $o(L_0)$ term is chosen so that C contains at least L_0 of M-cells with high probability. The cells outside of the cluster C may interact with the finite control only when $q \in Q_r$, which means that only Γ_A-parts may touch. This fact motivates the following representation of the initial state: Let $\boldsymbol{y}$ be the first $|C|$ components of $\boldsymbol{x}$, that is, the configuration of sites in the cluster C, and denote by Y the set of all possible $\boldsymbol{y}$'s. Then, the initial state of the

total system can be expressed as

$$|\psi_{V,L}\rangle = \sum_{\boldsymbol{y}\in Y} c_{\boldsymbol{y}} |\boldsymbol{y}\rangle \otimes (V|e_1\rangle)^{\otimes L-|C|}. \tag{S.64}$$

We call $\boldsymbol{y}$ "good" initial configurations if (i) TM1 correctly decode the input code $\boldsymbol{u}$ for TM2, and (ii) in the dynamics induced by the isometry U the finite control does not come out from the cluster C before TM2 reaches the halting state. We write as Y^* the set of good initial configurations in the above sense, and define the following unnormalized state:

$$|\psi^*_{V,L}\rangle := \sum_{\boldsymbol{y}\in Y^*} c_{\boldsymbol{y}} |\boldsymbol{y}\rangle \otimes (V|e_1\rangle)^{\otimes L-|C|}. \tag{S.65}$$

By construction, states outside Y^* have negligibly small probability weight, and thus for any $\delta > 0$ we can prepare $\left|\langle\psi_{V,L}|\psi^*_{V,L}\rangle\right| > 1-\delta$. The dynamics starting from $|\psi^*_{V,L}\rangle$ and $|\psi_{V,L}\rangle$ are arbitrarily close to each other because the trace norm between the states $|\psi^*_{V,L}(t)\rangle = e^{-iHt}|\psi^*_{V,L}\rangle$ and $|\psi_{V,L}(t)\rangle = e^{-iHt}|\psi_{V,L}\rangle$ is bounded as

$$\begin{aligned} \| \, |\psi^*_{V,L}(t)\rangle\langle\psi^*_{V,L}(t)| - |\psi_{V,L}(t)\rangle\langle\psi_{V,L}(t)| \, \|_1 =& \| \, |\psi^*_{V,L}\rangle\langle\psi^*_{V,L}| - |\psi_{V,L}\rangle\langle\psi_{V,L}| \, \|_1 \\ \leq& 2\sqrt{1-\left|\langle\psi_{V,L}|\psi^*_{V,L}\rangle\right|^2} \\ \leq& 2\sqrt{2\delta}. \end{aligned} \tag{S.66}$$

Since δ can be arbitrarily small, in the following we regard $|\psi^*\rangle$ as the initial state itself.

For each good initial configuration $\boldsymbol{y}$, we define the j-th state as

$$|j,\boldsymbol{y}\rangle := U^{j-1}|\boldsymbol{y}\rangle \otimes (V|e_1\rangle)^{\otimes L-|C|}. \tag{S.67}$$

Recalling that U updates the Γ-part of the M-cell in simulating TM1 and TM2, and Γ_A part after the halting, we find that U either changes $\boldsymbol{y}$ into another configuration $\boldsymbol{y}'$, or interchanges $V|e_k\rangle$'s $(k=1,2)$. The above observation implies that $|j,\boldsymbol{y}\rangle$ is written in the form of

$$|j,\boldsymbol{y}\rangle = |\boldsymbol{y}'\rangle \otimes (\otimes_{i=|C|+1}^{L} V|e_{k_i}\rangle), \tag{S.68}$$

where $k_i \in \{1,2\}$. This confirms that two states at different steps with the same initial state are orthogonal to each other:

$$\langle j,\boldsymbol{y}|j',\boldsymbol{y}\rangle = 0 \tag{S.69}$$

for any $j \neq j'$.

10.3 Case when TM2 halts 2: long-time average of $\mathcal{A}$

Using the eigenenergies and eigenstates shown in Eqs. (S.43) and (S.44), $e^{-iHt}\left|1,\boldsymbol{y}\right\rangle$ is computed as

$$\begin{aligned} e^{-iHt}\left|1,\boldsymbol{y}\right\rangle =&\sqrt{\frac{2}{J_{\boldsymbol{y}}+1}}\sum_{k=1}^{J_{\boldsymbol{y}}} e^{-iE_{k,\boldsymbol{y}}t}\sin\frac{k\pi}{J_{\boldsymbol{y}}+1}\left|E_{k,\boldsymbol{y}}\right\rangle \\ =&\frac{2}{J_{\boldsymbol{y}}+1}\sum_{k=1}^{J_{\boldsymbol{y}}} e^{-iE_{k,\boldsymbol{y}}t}\sin\frac{k\pi}{J_{\boldsymbol{y}}+1}\sum_{j=1}^{J_{\boldsymbol{y}}}\sin\frac{jk\pi}{J_{\boldsymbol{y}}+1}\left|j,\boldsymbol{y}\right\rangle, \end{aligned} \tag{S.70}$$

where

$$E_{k,\boldsymbol{y}} := 2\cos\frac{k\pi}{J_{\boldsymbol{y}}+1}, \tag{S.71}$$

$$\left|E_{k,\boldsymbol{y}}\right\rangle := \sqrt{\frac{2}{J_{\boldsymbol{y}}+1}}\sum_{j=1}^{J_{\boldsymbol{y}}}\sin\frac{jk\pi}{J_{\boldsymbol{y}}+1}\left|j,\boldsymbol{y}\right\rangle \tag{S.72}$$

are the k-th energy eigenvalue and corresponding energy eigenstate of the effective Hamiltonian $H_{\text{eff}} = \sum_j \left|j+1,\boldsymbol{y}\right\rangle\left\langle j,\boldsymbol{y}\right| + \text{c.c.}$, and $J_{\boldsymbol{y}}$ is the total number of steps for the termination of M_{G} starting from the configuration in the cluster C as $\boldsymbol{y}$.

Using Eq. (S.39), the long-time average of $\mathcal{A}$ from the initial state $\left|\psi^*_{V,L}\right\rangle$ given in Eq. (S.65) is calculated as

$$\overline{\mathcal{A}} = \sum_{k,k',\boldsymbol{y},\boldsymbol{y}'}\chi(E_{k,\boldsymbol{y}}=E_{k',\boldsymbol{y}'})c^*_{\boldsymbol{y}}c_{\boldsymbol{y}'}\sqrt{\frac{2}{J_{\boldsymbol{y}}+1}}\sqrt{\frac{2}{J_{\boldsymbol{y}'}+1}}\sin\frac{k\pi}{J_{\boldsymbol{y}}+1}\sin\frac{k'\pi}{J_{\boldsymbol{y}'}+1}\left\langle E_{k,\boldsymbol{y}}\right|\mathcal{A}_L\left|E_{k',\boldsymbol{y}'}\right\rangle, \tag{S.73}$$

where $\chi(E_{k,\boldsymbol{y}}=E_{k',\boldsymbol{y}'})$ takes 1 if $E_{k,\boldsymbol{y}}=E_{k',\boldsymbol{y}'}$ and takes zero otherwise. The contribution from the case of $\boldsymbol{y}=\boldsymbol{y}'$ (diagonal elements) has already been calculated and shown to be a finite amount in Sec. 9. We shall prove that the contribution to $\overline{\mathcal{A}}$ from the case of $\boldsymbol{y}\neq\boldsymbol{y}'$ (off-diagonal elements) is sufficiently small.

Due to the form of Eq. (S.71), the condition $E_{k,\boldsymbol{y}}=E_{k',\boldsymbol{y}'}$ implies

$$\frac{k}{k'} = \frac{J_{\boldsymbol{y}}+1}{J_{\boldsymbol{y}'}+1}. \tag{S.74}$$

Let G be the greatest common divisor of $J_{\boldsymbol{y}}+1$ and $J_{\boldsymbol{y}'}+1$, and define $k_0 := \frac{J_{\boldsymbol{y}}+1}{G}$ and $k'_0 := \frac{J_{\boldsymbol{y}'}+1}{G}$. Then, k and k' with Eq. (S.74) are expressed as $k=lk_0$ and $k'=lk'_0$ with $l=1,2,\ldots,G$, and thus

$$\frac{k}{J_{\boldsymbol{y}}+1} = \frac{k'}{J_{\boldsymbol{y}'}+1} = \frac{l}{G} \tag{S.75}$$

is satisfied. Hence, for any $\boldsymbol{y} \neq \boldsymbol{y}'$ we have

$$
\begin{aligned}
&\sum_{\substack{k,k' \\ E_{k,\boldsymbol{y}}=E_{k',\boldsymbol{y}'}}} \sin\frac{k\pi}{J_{\boldsymbol{y}}+1} \sin\frac{k'\pi}{J_{\boldsymbol{y}'}+1} \langle E_{k,\boldsymbol{y}}|\mathcal{A}_L|E_{k',\boldsymbol{y}'}\rangle \\
=&\sum_{l=1}^{G-1} \sin^2\frac{l\pi}{G} \langle E_{k_0 l,\boldsymbol{y}}|\mathcal{A}_L|E_{k_0' l,\boldsymbol{y}'}\rangle \\
=&\sqrt{\frac{2}{J_{\boldsymbol{y}}+1}}\sqrt{\frac{2}{J_{\boldsymbol{y}'}+1}} \sum_{l=1}^{G}\sum_{j=1}^{J_{\boldsymbol{y}}}\sum_{j'=1}^{J_{\boldsymbol{y}'}} \sin\frac{jl\pi}{G}\sin\frac{j'l\pi}{G}\sin^2\frac{l\pi}{G}\langle j,\boldsymbol{y}|\mathcal{A}_L|j',\boldsymbol{y}'\rangle \\
=&\frac{1}{2}\sqrt{\frac{2}{J_{\boldsymbol{y}}+1}}\sqrt{\frac{2}{J_{\boldsymbol{y}'}+1}} \sum_{l=1}^{G}\sum_{j=1}^{J_{\boldsymbol{y}}}\sum_{j'=1}^{J_{\boldsymbol{y}'}} \left(\cos\frac{(j+j')l\pi}{G}+\cos\frac{(j-j')l\pi}{G}\right)\sin^2\frac{l\pi}{G}\langle j,\boldsymbol{y}|\mathcal{A}_L|j',\boldsymbol{y}'\rangle \\
\leq&\frac{1}{2}\sqrt{\frac{2}{J_{\boldsymbol{y}}+1}}\sqrt{\frac{2}{J_{\boldsymbol{y}'}+1}} \sum_{l=1}^{G}\sum_{j=1}^{J_{\boldsymbol{y}}}\sum_{j'=1}^{J_{\boldsymbol{y}'}} \left(\cos\frac{(j+j')l\pi}{G}+\cos\frac{(j-j')l\pi}{G}\right)\langle j,\boldsymbol{y}|\mathcal{A}_L|j',\boldsymbol{y}'\rangle \\
\leq&\frac{1}{2}\sqrt{\frac{2}{J_{\boldsymbol{y}}+1}}\sqrt{\frac{2}{J_{\boldsymbol{y}'}+1}}(J_{\boldsymbol{y}}+1)(J_{\boldsymbol{y}'}+1)\langle j,\boldsymbol{y}|\mathcal{A}_L|j',\boldsymbol{y}'\rangle \\
\leq&\sqrt{(J_{\boldsymbol{y}}+1)(J_{\boldsymbol{y}'}+1)}\frac{\|A\|}{L}.
\end{aligned}
\tag{S.76}
$$

In the sixth line, we used the fact that $\sum_{l=1}^{G}\cos\frac{(j+j')l\pi}{G}$ is equal to G if $j+j'$ is a multiple of $2G$ and is equal to zero otherwise. In the seventh line, we used the following relation similar to Eq. (S.48)

$$
|\langle j,\boldsymbol{y}|\mathcal{A}_L|j',\boldsymbol{y}'\rangle| = \left|\frac{1}{L}\sum_{i=1}^{L}\langle j,\boldsymbol{y}|A_i|j',\boldsymbol{y}'\rangle\right| \leq \frac{\|A\|}{L} \tag{S.77}
$$

for any $\boldsymbol{y} \neq \boldsymbol{y}'$. As is the case of Eq. (S.48), this relation follows from the fact that the configurations $(j,\boldsymbol{y})$ and $(j',\boldsymbol{y}')$ differs at least a single site.

Substituting Eq. (S.76) into Eq. (S.73) in case of $\boldsymbol{y} \neq \boldsymbol{y}'$, we arrive at the upper bound for the off-diagonal sum:

$$
\begin{aligned}
&\left|\sum_{\substack{k,k',\boldsymbol{y},\boldsymbol{y}' \\ \boldsymbol{y}\neq\boldsymbol{y}'}} \chi(E_{k,\boldsymbol{y}}=E_{k',\boldsymbol{y}'})c_{\boldsymbol{y}}^* c_{\boldsymbol{y}'}\sqrt{\frac{2}{J_{\boldsymbol{y}}+1}}\sqrt{\frac{2}{J_{\boldsymbol{y}'}+1}}\sin\frac{k\pi}{J_{\boldsymbol{y}}+1}\sin\frac{k'\pi}{J_{\boldsymbol{y}'}+1}\langle E_{k,\boldsymbol{y}}|\mathcal{A}_L|E_{k',\boldsymbol{y}'}\rangle\right| \\
\leq&\frac{2\|A\|}{L}\sum_{\boldsymbol{y}\neq\boldsymbol{y}'}\left|c_{\boldsymbol{y}}^* c_{\boldsymbol{y}'}\right| \leq \frac{2\|A\|}{L}\left(\sum_{\boldsymbol{y}}|c_{\boldsymbol{y}}|\right)^2 \leq \frac{2\|A\|}{L}|Y^*|.
\end{aligned}
\tag{S.78}
$$

Notably, $|Y^*|$ is independent of the total system size L, and hence, by taking the thermodynamic limit $L\to\infty$ the right-hand side becomes arbitrarily small. Thus, using the result in Sec. 9, we have

$$
\overline{\mathcal{A}} \geq \frac{1}{4}\langle e_2|A|e_2\rangle - \alpha\|A\| \tag{S.79}
$$

and

$$\begin{aligned}\overline{\mathcal{V}\mathcal{A}\mathcal{V}^\dagger} \geq& \frac{1}{4}\langle e_2|VAV^\dagger|e_2\rangle - \alpha\|A\| \\ =& \frac{1}{4}(\sqrt{\alpha}\,|s_0, a_k\rangle\,\langle\psi_{\rm in}| + \sqrt{1-\alpha}\,\langle e_k|)A(\sqrt{\alpha}\,|s_0, a_k\rangle\,|\psi_{\rm in}\rangle + \sqrt{1-\alpha}\,|e_k\rangle) - \alpha\|A\| \\ \geq& \frac{1-\alpha}{4}\langle e_2|A|e_2\rangle - 2(\alpha + \sqrt{\alpha(1-\alpha)})\|A\|. \end{aligned} \tag{S.80}$$

By taking α sufficiently small, we arrive at the desired result

$$\min\{\overline{\mathcal{A}}, \overline{V\mathcal{A}V^\dagger}\} \geq \frac{1-\alpha}{4}\langle e_2|A|e_2\rangle - 2(\alpha + \sqrt{\alpha(1-\alpha)})\|A\| \geq \left(\frac{1}{4} - \eta\right)\langle e_2|A|e_2\rangle \tag{S.81}$$

for any $\eta > 0$.

10.4 Case when TM2 does not halt

In this subsection, we consider the case that TM2 does not halt with the input $\boldsymbol{u}$. To bound the off-diagonal elements from above, we need a completely different treatment from the case of halting, because the size of the cluster C now becomes the entire system (The cluster C should contain the working space of TM2, which is unlimited in case of non-halting).

To treat the non-halting case, we focus on the fact that most of the cells in the initial state are A-cells. By construction, the number of A-cells is invariant under the time evolution, and if the decoding of input succeeds, all of A-cells are filled with the symbol a_1 at all times.

We first restrict the state space to "good" configurations of $\boldsymbol{x}$. In this subsection, we employ the word "good" initial configurations with a slightly different definition from the previous subsection. We say that $\boldsymbol{x}$ is a "good" initial configuration if (i) the input for TM2 is correctly decoded by TM1, and (ii) the fraction of A-cells is larger than $1-\alpha'$ where α' is set properly as slightly larger than but close to α. We do not require the size of the working space for TM2. We write as X^* the set of good initial configurations in the above sense, and define the following unnormalized state:

$$|\psi_L^{**}\rangle := \sum_{\boldsymbol{x}\in X^*} c_{\boldsymbol{x}}\,|\boldsymbol{x}\rangle\,. \tag{S.82}$$

For a similar reason to the case of Y^* in Sec. 10.2, the dynamics starting from $|\psi_L^{**}\rangle$ denoted by $|\psi_L^{**}(t)\rangle$ is arbitrarily close to the actual dynamics $|\psi_L(t)\rangle$. Therefore, in the following, we consider the state $|\psi_L^{**}(t)\rangle$ instead of the actual one.

Let $P := |e_1\rangle\langle e_1|$ be the projector onto the A-cell filled with the symbol a_1, and P_i be the aforementioned projector acting on the i-th site. If $|\boldsymbol{x}\rangle$ represents a single configuration of $M_{\rm G}$, ${\rm Tr}[P_i\,|\boldsymbol{x}\rangle\langle\boldsymbol{x}|]$ equals 1 (resp, 0) iff the i-th cell is (resp. is not) an A-cell with the symbol a_1. Hence, we have

$${\rm Tr}\left[\sum_{i=1}^{L+1} P_i\,|\psi_L^{**}(t)\rangle\langle\psi_L^{**}(t)|\right] \geq (1-\alpha')L \tag{S.83}$$

for any t. Let $\rho_i(t)$ denote the reduced density operator of $|\psi_L^{**}(t)\rangle\langle\psi_L^{**}(t)|$ onto the i-th

site, and define the averaged density operator over all sites as

$$\overline{\rho}(t) := \frac{1}{L}\sum_{i=1}^{L} \rho_i(t). \tag{S.84}$$

Then, using a relation

$$\mathrm{Tr}[P\overline{\rho}(t)] = \langle e_1|\overline{\rho}(t)|e_1\rangle \geq 1-\alpha', \tag{S.85}$$

we arrive at the desired result:

$$\begin{aligned} |\langle\psi_L(t)|\mathcal{A}_L|\psi_L(t)\rangle| &= |\langle\psi_L(t)|\mathcal{A}_L|\psi_L(t)\rangle - \langle e_1|A|e_1\rangle| \\ &= |\mathrm{Tr}[A\overline{\rho}(t)] - \langle e_1|A|e_1\rangle| \\ &\leq \|\overline{\rho}(t) - |e_1\rangle\langle e_1|\,\|_1\|A\| \\ &\leq 2\sqrt{1-\frac{(1-\alpha')L}{L+1}}\|A\| \\ &< 2\sqrt{\alpha'}\|A\|. \end{aligned} \tag{S.86}$$

Since we can set $\alpha' < 2\alpha$, we arrive at the desired relation $\overline{\mathcal{A}} < 2\sqrt{2\alpha}\|A\|$.

To show Lemma 1, we also need to evaluate the state with V rotation, which is evaluated as

$$\begin{aligned} &\left|\langle\psi_L|\mathcal{V}\mathcal{A}_L\mathcal{V}^\dagger|\psi_L\rangle - \langle e_1|A|e_1\rangle\right| \\ \leq &\left|\langle\psi_L|\mathcal{V}\mathcal{A}_L\mathcal{V}^\dagger|\psi_L\rangle - \langle e_1|VAV^\dagger|e_1\rangle\right| + \left|\langle e_1|VAV^\dagger|e_1\rangle - \langle e_1|A|e_1\rangle\right|. \end{aligned} \tag{S.87}$$

The first term has readily obtained through Eq. (S.86) with replacing A to $VAV^\dagger$, which bounds the first term from above by $2\sqrt{2\alpha}\|A\|$. The second term is calculated as

$$\begin{aligned} \left|\langle e_1|VAV^\dagger|e_1\rangle - \langle e_1|A|e_1\rangle\right| &\leq \|A\|\|\,|e_1\rangle\langle e_1| - V^\dagger|e_1\rangle\langle e_1|V\| \\ &= \|A\|\sqrt{1-\langle e_1|V|e_1\rangle} \\ &= \|A\|\sqrt{\alpha}, \end{aligned} \tag{S.88}$$

where in the last line we used Eqs. (S.56) and (S.55). Combining them, we find

$$\max\{\overline{\mathcal{A}}, \overline{V\mathcal{A}V^\dagger}\} \leq 4\sqrt{\alpha}\|A\|, \tag{S.89}$$

whose right-hand side can become arbitrarily small by taking α sufficiently small. In particular, $\max\{\overline{\mathcal{A}}, \overline{V\mathcal{A}V^\dagger}\} \leq \eta$ is fulfilled, which completes the whole proof of Lemma 1.

10.5 Note: dimension of the local Hilbert space

We have not discussed how large the sufficient dimension of the local Hilbert space is. We here present a rough estimation of it instead of rigorous calculation.

To reduce the dimension, it is useful to enlarge the tape alphabet. More symbols the tape alphabet has, fewer states the finite control needs to have. It has been established that there are URTMs with 10-state and 8-symbol, 15-state and 6-symbol, 24-state and 4-symbol, 32-state and 3-symbol, and 138-state and 2-symbol [10]. By employing the 24-state 4-symbol URTM, 24 states in the finite control and $(4+1)\times 2\times 2^2 = 40$ symbols in the

tape alphabet suffice to simulate TM2 (see also Supplementary Figure 3). Although we do not evaluate the sufficient number of states and symbols for TM1, since the tasks of TM1 are all elementary; counting the number of 1's and 0's. computing logarithm, and division, we roughly estimate that 50 states and 4 symbols suffice for TM1. If the above estimation is correct, the sufficient dimension of the local Hilbert space is $(24+50+1)+(40+2+2) = 119$.

11 Proof of Theorem 2 (undecidability of thermalization)

Our main idea to prove Theorem 2 is tuning the equilibrium value $\mathrm{Tr}[\mathcal{A}\rho^{\mathrm{MC}}]$ to the desired target value A^* by changing the Hamiltonian. To this end, we need some refinements and modifications of the proof of Lemma 1. In Sec.11.1, we first specify the long-time average of $\mathcal{A}$ when the URTM halts. In Sec.11.2, we modify the dynamics of TM3, which enables us to push the value of $\overline{\mathcal{A}}$ in case of halting away from that in case of non-halting. In Sec.11.3, we construct a family of Hamiltonians whose microcanonical average of $\mathcal{A}$ is tuned to the target value A^*.

11.1 Value of $\overline{\mathcal{A}}$ in thermodynamic limit when TM2 halts

In the previous section, we only derive an inequality for the value of $\overline{\mathcal{A}}$ when TM2 halts. We here briefly calculate the value of $\overline{\mathcal{A}}$ in the thermodynamic limit when TM2 halts.

We start from Eq. (S.50) and the equality part of Eq. (S.51) for non-uniform (computational basis) initial states. By taking L to infinity, the number of steps before TM2 halts is negligibly small compared to the total number of steps J. Thus the right-hand side in the equality of Eq. (S.51) is calculated as

$$\lim_{L\to\infty} \frac{3}{2(J+1)} \left(\langle \boldsymbol{x}^1|\mathcal{A}_L|\boldsymbol{x}^1\rangle + \langle \boldsymbol{x}^J|\mathcal{A}_L|\boldsymbol{x}^J\rangle\right) + \frac{1}{J+1}\sum_{j=2}^{J-1} \langle \boldsymbol{x}^j|\mathcal{A}_L|\boldsymbol{x}^j\rangle = \frac{1-\alpha}{2}\langle e_2|A|e_2\rangle \tag{S.90}$$

for computational basis initial states, where we assumed that M-cells and A-cells are uniformly distributed. By combining Eq. (S.50), this relation leads to the bound for $\overline{\mathcal{A}}$ for computational basis initial states: $\left|\overline{\mathcal{A}} - \frac{1-\alpha}{2}\langle e_2|A|e_2\rangle\right| \leq \alpha\|A\|$. In the case of uniform initial states (excepts for the first site), we have already shown that off-diagonal terms and various other correction terms vanish in the thermodynamic limit. Thus, by using Eqs. (S.73) and (S.78), we readily have

$$\left|\overline{\mathcal{A}} - \frac{1-\alpha}{2}\langle e_2|A|e_2\rangle\right| \leq \alpha\|A\|, \tag{S.91}$$

$$\left|\overline{V\mathcal{A}V^\dagger} - \frac{1-\alpha}{2}\langle e_2|A|e_2\rangle\right| \leq 2(\alpha + \sqrt{\alpha(1-\alpha)})\|A\|, \tag{S.92}$$

for uniform initial states. This means that both $\overline{\mathcal{A}}$ and $\overline{V\mathcal{A}V^\dagger}$ converge close to $\frac{1-\alpha}{2}\langle e_2|A|e_2\rangle$ in the thermodynamic limit with errors $\alpha\|A\|$ and $2(\alpha+\sqrt{\alpha(1-\alpha)})\|A\|$. Since α can be taken arbitrarily small, these relations roughly state that both $\overline{\mathcal{A}}$ and $\overline{V\mathcal{A}V^\dagger}$ converge around $\frac{1}{2}\langle e_2|A|e_2\rangle$ with arbitrarily small errors.

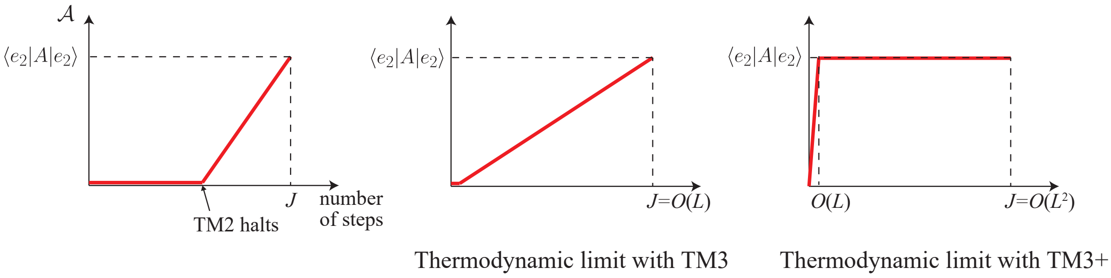

Supplementary Figure 5: (Left): A typical graph of the behavior of the expectation value of A inf the classical TM (with TM1, TM2, and TM3) for the number of steps. After TM2 halts, the expectation value of A starts to increase and reaches $\langle e_2|A|e_2\rangle$ at the final step J. (Middle): Thermodynamic limit of the left graph. Since TM2 halts in constant steps, in $L \to \infty$ limit the dynamics of TM3 dominates this graph. (Right): Thermodynamics limit when we use TM3+ instead of TM3. After flipping all A-cells from a_1 to a_2, TM3+ runs for around $L^2/2$ steps, which makes the long-time average $\overline{\mathcal{A}}$ arbitrarily close to $\langle e_2|A|e_2\rangle$.

11.2 Pushing the value of $\overline{\mathcal{A}}$ away from zero

In the setup in the proof of Lemma 1, the value of $\overline{\mathcal{A}}$ when TM2 halts is shown to approach $\frac{1}{2}\langle e_2|A|e_2\rangle$. This value, more precisely $\frac{1}{2}(\langle e_1|A|e_1\rangle + \langle e_2|A|e_2\rangle)$, comes from the following calculation: In the thermodynamic limit, the time evolution of $A(t)$ in the classical TM is roughly described by a linear function in time, and the dynamics stops when $A(t)$ reaches $\langle e_2|A|e_2\rangle$ (see the middle graph in Supplementary Figure 5). Thus, the time average of $\mathcal{A}(t)$ is $\frac{1}{2}\langle e_2|A|e_2\rangle$.

In order to inflate $\overline{\mathcal{A}}$, we modify the rule of TM3 such that the machine spends most of the time with $\mathcal{A}(t)$ at $\langle e_2|A|e_2\rangle$. The modified TM3, named TM3+, has two additional states in A-cells, and three additional internal states in the finite control. We first recall the states and the rule of TM3[10]: In TM3, A-cells take two different states, a_1 and a_2, and the finite control has a single internal state r. The rule of the dynamics is as follows:

- If the head reads a_1 with the internal state r, then the state of the A-cell is changed to a_2 and the finite control moves right.
- If the head reads a_2 with the internal state r, then the finite control stops.

Now, we describe the states and the rule of TM3+. In TM3+, A-cells take four different states, a_1, a_2, b_{l} and b_{r}, and the finite control has four different internal states, r, r_{l}, r_{r}, and r_{c}. The rule of the dynamics is as follows:

- If the head reads a_1 with the internal state r, then the state of the A-cell is changed to a_2, and the finite control moves right.
- If the head reads a_2 with the internal state r, then the state of the A-cell is changed to b_{l}, the internal state is changed to r_{c}, and the finite control moves right.
- If the head reads a_2 with the internal state r_{c}, then the state of the A-cell is changed to b_{r}, the internal state is changed to r_{l}, and the finite control does not move (state 1-2 to 1-3 in Supplementary Figure 6).

[10]Since TM3 passes M-cells, we omit the description on M-cells.

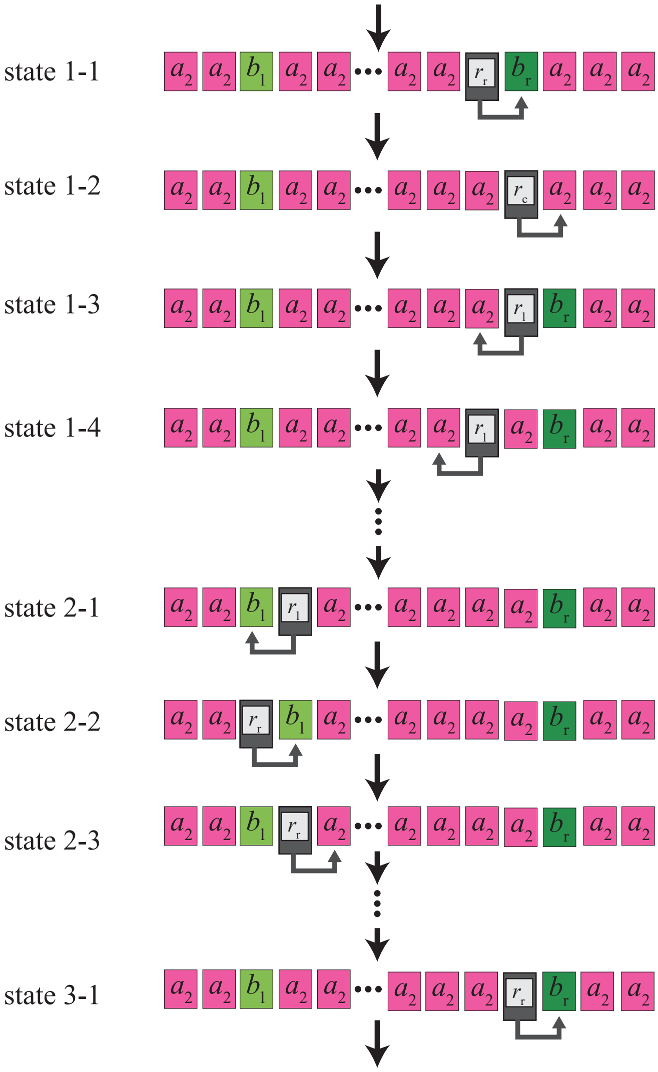

Supplementary Figure 6: Schematic of the dynamics of TM3+. We drop M-cells for brevity. The states 1-1 and 3-1 are very similar, but the cell with $b_{\rm r}$ moves right one cell.

- If the head reads a_2 with the internal state $r_{\rm l}$, then the state of the A-cell and the internal state are kept unchanged, and the finite control moves left (state 1-3 to1-4).
- If the head reads $b_{\rm l}$ with the internal state $r_{\rm l}$, then the state of the A-cell is kept unchanged, the internal state is changed to $r_{\rm r}$, and the finite control moves left (state 2-1 to 2-2).
- If the head reads a_2 or $b_{\rm l}$ with the internal state $r_{\rm r}$, then the state of the A-cell and the internal state is kept unchanged, and the finite control moves right (state 2-2 to 2-3 and 2-3 to 3-1).
- If the head reads $b_{\rm r}$ with the internal state $r_{\rm r}$, then the state of the A-cell is changed to a_2, the internal state is changed to $r_{\rm c}$, and the finite control moves right (state 1-1 to 1-2).
- If the head reads $b_{\rm l}$ with the internal state $r_{\rm c}$, then the finite control stops.

This rule yields the following dynamics: After flipping all A-cells to a_2, then TM3+ shuttles between $b_{\rm l}$ and $b_{\rm r}$ with pushing $b_{\rm r}$. AT the beginning of this shuttle, the cell $b_{\rm r}$ sits the right of $b_{\rm l}$. During a single shuttle between $b_{\rm l}$ and $b_{\rm r}$, $b_{\rm l}$ does not move on average and $b_{\rm r}$ moves right one cell (see Supplementary Figure 6). This shuttle finishes when $b_{\rm r}$ hits $b_{\rm l}$ from the left. The flipping from a_1 to a_2 takes $O(L)$ steps, while the shuttle process after flipping takes $O(L^2)$ steps. Namely, at almost all times, all A-cells take the state a_2. Thus, following the argument in the previous subsection, we find that in the corresponding quantum system the value of $\overline{\mathcal{A}}$ approaches $\langle e_2|A|e_2\rangle$ when TM2 halts.

11.3 Tuning the microcanonical average

We now demonstrate how to tune the long-time average $\overline{\mathcal{A}}$ in case of halting to the microcanonical average $\mathrm{Tr}[\mathcal{A}\rho^{\rm MC}]$. As seen in the previous subsections, by employing TM3+ the long-time average $\overline{\mathcal{A}}$ in case of halting (resp. non-halting) approaches $\langle e_2|A|e_2\rangle$ (resp. $\langle e_1|A|e_1\rangle = 0$) in the thermodynamic limit. We shall show the existence of a proper orthonormal set[11] $\{|e_i\rangle\}$ where $\mathrm{Tr}[\mathcal{A}\rho^{\rm MC}] = \langle e_2|A|e_2\rangle$ holds.

We introduce the Hilbert subspace with states orthogonal to $|\phi_0\rangle$, $|\phi_1\rangle$, $A\,|\phi_0\rangle$ and $A\,|\phi_1\rangle$ denoted by $\mathcal{S} \subset \mathcal{H}$. Let $P_\mathcal{S}$ be the projection operator onto $\mathcal{S}$. We consider an operator $P_\mathcal{S} A P_\mathcal{S}$ on $\mathcal{S}$, and express its eigenstates as $\{|\sigma_i\rangle\}$. Since $P_\mathcal{S}\,|\sigma_i\rangle = |\sigma_i\rangle$ and $P_\mathcal{S}\,|\sigma_j\rangle = |\sigma_j\rangle$, we have

$$\langle \sigma_i|A|\sigma_j\rangle = 0 \quad (i \neq j). \tag{S.93}$$

We set the indices of $\{|\sigma_i\rangle\}$ in decreasing order of expectation value of A;

$$\langle \sigma_1|A|\sigma_1\rangle \geq \langle \sigma_2|A|\sigma_2\rangle \geq \cdots \geq \langle \sigma_{d'}|A|\sigma_{d'}\rangle\,, \tag{S.94}$$

where we denoted the dimension of $\mathcal{S}$ by d'. Thanks to the assumption $\langle \phi_2|A|\phi_2\rangle > \max_{|\psi\rangle\in\mathrm{span}\{|\phi_0\rangle,|\phi_1\rangle\}} \langle\psi|A|\psi\rangle$ and $\langle \phi_3|A|\phi_3\rangle < \min_{|\psi\rangle\in\mathrm{span}\{|\phi_0\rangle,|\phi_1\rangle\}} \langle\psi|A|\psi\rangle$, we find[12]

$$\langle \sigma_1|A|\sigma_1\rangle > \max_{|\psi\rangle\in\mathrm{span}\{|\phi_0\rangle,|\phi_1\rangle\}} \langle\psi|A|\psi\rangle\,, \tag{S.95}$$

$$\langle \sigma_{d'}|A|\sigma_{d'}\rangle < \min_{|\psi\rangle\in\mathrm{span}\{|\phi_0\rangle,|\phi_1\rangle\}} \langle\psi|A|\psi\rangle\,. \tag{S.96}$$

Let $\rho_1^{\rm MC}$ be the reduced density matrix of a microcanonical state to a single site with energy $\langle\psi_0|H|\psi_0\rangle$ ($|\psi_0\rangle := |\phi_0\rangle\otimes|\phi_1\rangle\otimes\cdots\otimes|\phi_1\rangle$). Due to the translation invariance of H, $\mathrm{Tr}[\mathcal{A}\rho^{\rm MC}] = \mathrm{Tr}[A\rho_1^{\rm MC}]$ is satisfied. Note that the Hamiltonian H depends on the input code $\boldsymbol{u}$ and several parameters, and thus $\rho_1^{\rm MC}$ also depends on them. We formally expand it with the set $\{|e_i\rangle\}$ as $\rho_1^{\rm MC} = \sum_{i,j} |e_i\rangle\langle e_j|\,\langle e_i|\rho_1^{\rm MC}|e_j\rangle =: \sum_{i,j}|e_i\rangle\langle e_j|\,\rho_{ij}^{\rm MC}$. We have already set $|e_0\rangle = |\phi_0\rangle$ and $|e_1\rangle = |\phi_1\rangle$.

We first consider the case that $\langle\sigma_2|A|\sigma_2\rangle \neq \langle\sigma_{d'-1}|A|\sigma_{d'-1}\rangle$. We set the orthonormal set

[11] This set is not CONS because this set does not span the whole Hilbert space. However, since the dynamics are closed in this subspace spanned by this set, this point does not matter to our argument. (We assign extremely large energy to states outside this subspace in order to keep the contribution from these states to microcanonical average negligible).

[12] Since $|\phi_2\rangle \in \mathcal{S}$, we can expand $|\phi_2\rangle$ as $|\phi_2\rangle = \sum_i c_i\,|\sigma_i\rangle$. Due to the diagonal property of A with the orthonormal set $\{|\sigma_i\rangle\}$, $\langle\phi_2|A|\phi_2\rangle = \sum_i |c_i|^2\,\langle\sigma_i|A|\sigma_i\rangle < \langle\sigma_1|A|\sigma_1\rangle$. A similar argument holds for $|\phi_3\rangle$.

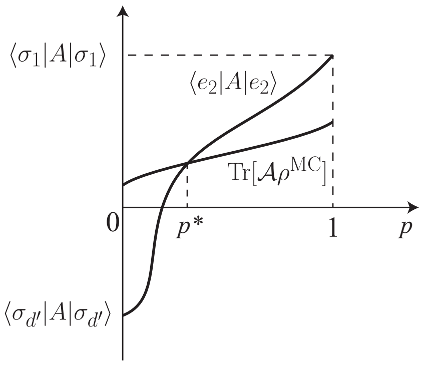

Supplementary Figure 7: Tuning of p from $p = 0$ to $p = 1$. Owing to $\langle\sigma_1|A|\sigma_1\rangle > \mathrm{Tr}[A\rho_1^{\mathrm{MC}}] > \langle\sigma_{d'}|A|\sigma_{d'}\rangle$, there must exist a proper p (denoted by p^* in this graph) with which $\langle e_2|A|e_2\rangle = \mathrm{Tr}[A\rho_1^{\mathrm{MC}}]$ is satisfied.

$\{|e_i\rangle\}$ as

$$|e_2\rangle = \sqrt{p}\,|\sigma_1\rangle + \sqrt{1-p}\,|\sigma_{d'}\rangle\,, \tag{S.97}$$

$$|e_3\rangle = \sqrt{q}\,|\sigma_2\rangle + \sqrt{1-q}\,|\sigma_{d'-1}\rangle\,, \tag{S.98}$$

$$|e_i\rangle = |\sigma_{i-1}\rangle\,, \tag{S.99}$$

with $i \geq 4$. By permuting the label of $\{|e_i\rangle\}$ if necessary, without loss of generality we suppose that the diagonal elements of $|e_3\rangle$ in the reduced microcanonical state is nonzero: $\rho_{33}^{\mathrm{MC}} \neq 0$. Since all off-diagonal elements of A with this orthonormal set is zero, the microcanonical average reads

$$\mathrm{Tr}[A\rho_1^{\mathrm{MC}}] = \sum_{i=2} \langle e_i|A|e_i\rangle\,\rho_{ii}^{\mathrm{MC}} + \langle e'|A|e'\rangle\,\langle e'|\rho_1^{\mathrm{MC}}|e'\rangle + \langle e''|A|e''\rangle\,\langle e''|\rho_1^{\mathrm{MC}}|e''\rangle\,, \tag{S.100}$$

where $|e'\rangle\,, |e''\rangle \in \mathcal{T} := \mathrm{span}\{|\phi_0\rangle\,, |\phi_1\rangle\}$ are proper states diagonalizing $P_{\mathcal{T}}AP_{\mathcal{T}}$ in this subspace $\mathcal{T}$. By construction, for any p and q,

$$\langle\sigma_1|A|\sigma_1\rangle > \mathrm{Tr}[A\rho_1^{\mathrm{MC}}] > \langle\sigma_{d'}|A|\sigma_{d'}\rangle \tag{S.101}$$

is satisfied.

We now change the parameter p from $p = 0$ to $p = 1$ continuously with a fixed q. Accordingly the long-time average $\langle e_2|A|e_2\rangle$ changes from $\langle\sigma_{d'}|A|\sigma_{d'}\rangle$ to $\langle\sigma_1|A|\sigma_1\rangle$ continuously. Hence, there exists a proper $0 \leq p^* \leq 1$ which fulfills $\langle e_2|A|e_2\rangle = \mathrm{Tr}[A\rho_1^{\mathrm{MC}}]$ (see Supplementary Figure 7). Note that by tuning q if needed, we can safely avoid the undesired situation that the above coincidence happens at $\mathrm{Tr}[A\rho_1^{\mathrm{MC}}] = \langle e_2|A|e_2\rangle = 0$. In summary, by employing the above p^*, these orthonormal set $\{|e_i\rangle\}$ realizes the Hamiltonian with which the long-time average $\overline{\mathcal{A}}$ is equal to $\mathrm{Tr}[A\rho_1^{\mathrm{MC}}] \neq 0$ if and only if the URTM with the input $\boldsymbol{u}$ halts.

We next consider the case that $\langle\sigma_2|A|\sigma_2\rangle = \langle\sigma_3|A|\sigma_3\rangle = \cdots = \langle\sigma_{d'-1}|A|\sigma_{d'-1}\rangle \neq$

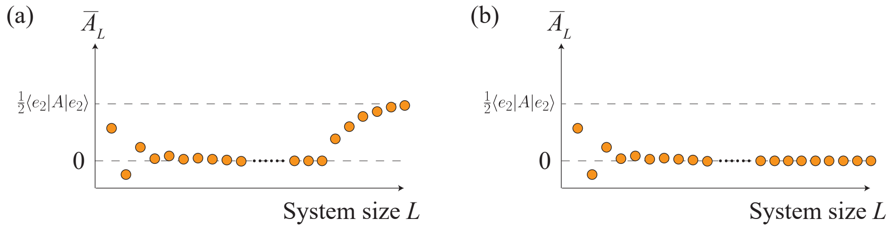

Supplementary Figure 8: A possible situation in numerical exact diagonalization. Although the long-time average $\overline{\mathcal{A}}_L$ appears to converge to some value, it may suddenly deviate from this value and converge to another value as (a). We cannot distinguish this long metastable case (a) from a true convergence case (b).

$\langle\sigma_1|A|\sigma_1\rangle$ [13]. In this case, we set

$$|e_2\rangle = \sqrt{p}(\sqrt{q}\,|\sigma_1\rangle - \sqrt{1-q}\,|\sigma_2\rangle) + \sqrt{1-p}\,|\sigma_{d'}\rangle \tag{S.102}$$

$$|e_3\rangle = \sqrt{1-q}\,|\sigma_1\rangle + \sqrt{q}\,|\sigma_2\rangle \tag{S.103}$$

$$|e_i\rangle = |\sigma_{i-1}\rangle \tag{S.104}$$

with $i \geq 4$, where we again supposed $\rho_{33}^{\mathrm{MC}} \neq 0$ without loss of generality. If q is close to 1, we have $\langle e_2|A|e_2\rangle > \mathrm{Tr}[A\rho_1^{\mathrm{MC}}]$ by setting $p = 1$. Thus, by changing p from $p = 0$ to $p = 1$, we again find that there exists a proper p^* such that $\langle e_2|A|e_2\rangle = \mathrm{Tr}[A\rho_1^{\mathrm{MC}}]$ is satisfied. We can avoid the unwanted situation $\mathrm{Tr}[A\rho_1^{\mathrm{MC}}] = \langle e_2|A|e_2\rangle = 0$ by tuning q. In summary, by employing the above p^*, these orthonormal set $\{|e_i\rangle\}$ realizes the Hamiltonian with which the long-time average $\overline{\mathcal{A}}$ is equal to $\mathrm{Tr}[A\rho_1^{\mathrm{MC}}] \neq 0$ if and only if the URTM with the input $\boldsymbol{u}$ halts. This completes the proof of Theorem 2.

12 Remarks on the infinite limit

12.1 What happens if we numerically simulate this system?

Our result shows that the long-time average of $\mathcal{A}$ is uncomputable even with the unlimited computational resource. Therefore, one may wonder about the relation between the uncomputability and the fact that we can numerically simulate any system with finite size. We here comment on this point.

Although there are several possible scenarios, we here describe the most intuitive one. We consider the case that TM2 halts after very long steps. If the system size is insufficient, the finite control passes the periodic boundary before halting and stops its movement. With such a small system size, $\overline{\mathcal{A}}_L$ takes a value close to zero. On the other hand, if the system size becomes sufficiently large for TM2 to halt, then $\overline{\mathcal{A}}_L$ start approaching around $\frac{1}{2}\langle e_2|A|e_2\rangle$. The problem lies in the fact that we never know what system size is *sufficiently large.* The undecidability of Turing machines tells that we cannot exclude the possibility that a Turing machine runs for very long steps and suddenly halts. In terms of numerical simulations, any large numerical simulation might be fooled due to insufficient system size, and thus even with the unlimited computational resource we cannot distinguish the case with a long

[13] In case of $\langle\sigma_1|A|\sigma_1\rangle = \langle\sigma_2|A|\sigma_2\rangle = \cdots = \langle\sigma_{d'-2}|A|\sigma_{d'-2}\rangle$, by reversing the role of $|\sigma_1\rangle$ and $|\sigma_{d'}\rangle$ a similar argument holds.

metastable state (Supplementary Figure 8 (a)) and the case that the system has already relaxed (Supplementary Figure 8 (b)). This shows clear contrast to the numerical simulation of conventional many-body systems, where by taking sufficiently large system size we can make the amount of error from the true value in the thermodynamic limit arbitrarily small.

12.2 Difference from the behavior of near-integrable systems

The above explanation may convey the impression that our constructed system is essentially the same as near-integrable systems; $H = H_{\rm int} + \varepsilon V$, with an integrable Hamiltonian $H_{\rm int}$ and a small perturbation εV. In near-integrable systems, the small parameter ε characterizes the necessary system size to reach the true long-time average, which can become extremely large by taking ε close to zero. By preparing a sufficiently large system, where its sufficiency is characterized by ε, we succeed in reaching the correct long-time average within an arbitrarily small error.

We claim that our system does not have such a small parameter going to an arbitrarily small number, and as its consequence sufficiently large system size does not exist. One may feel that the length of an input code $\boldsymbol{u}$, which induces the change in the input Hamiltonian H, serves as a small parameter. If this guess is true, by preparing a large system whose size is determined by the input length, we safely observe the presence or absence of thermalization in this large system. However, unfortunately, this is not the case.

To explain why, we introduce the *busy beaver function* BB(n), which is the maximum number of steps taken by a *halting* Turing machine (TM) with n different internal states and an empty input code. We remark that a TM with n internal states and an empty input can be implemented by a fixed URTM with a small number of internal states and an input code with length at most $l(n)$, and vice versa. Thus, the busy beaver function can be regarded as the maximum number of steps the URTM can take with a halting input code with a given length. This fact directly implies that the estimation of the sufficient size for thermalization with a given input code $\boldsymbol{u}$ is essentially the same as the computation of the busy beaver function.

However, the busy beaver function is proven not to be a computable function. More surprisingly, it is also proven that we cannot compute the busy beaver function BB(n) with $n \geq 748$ as far as the Zermelo-Fraenkel set theory with the axiom of choice (ZFC), which is roughly equivalent to our mathematics, is consistent [11,12]. This striking result comes from the fact that there exists a 748-state TM which halts if and only if the ZFC is inconsistent. The Gödel's incompleteness theorem reveals that the ZFC cannot prove the consistency of the ZFC itself. Hence, if BB(748) is computable, then by running the above TM for ZFC consistency until the step determined by BB(748) we can prove the consistency of the ZFC, which is a contradiction.

The incomputability of BB(748) leads to the absence of small parameters in our system of thermalization. We collect all Hamiltonians which correspond to the TMs with 748 internal states and empty input codes. Since there are only a finite number of Hamiltonians, any two Hamiltonians in this set have a finite difference between them (i.e., there is no small parameter going to zero). However, as we have seen, we do not have *sufficiently large system size* in spite of no small parameter in these Hamiltonians, as long as our mathematics is consistent.

Supplementary References

[1] A. M. Turing, *On computable numbers, with an application to the Entscheidungsproblem.* Proc. London Math. Soc. **42**, 230 (1937).

[2] C. Moore and S. Mertens, *Nature of computation.* Oxford university press (2011).

[3] R. Feynman, *Quantum mechanical computers.* Optics News **11**, 11 (1985).

[4] A. Yu. Kitaev, A.H. Shen, and M.N. Vyalyi. *Classical and Quantum Computation.* Vol. 47 of Graduate Studies in Mathematics. American Mathematical Society, (2002).

[5] P. Bocchieri and A. Loinger, *Quantum Recurrence Theorem.* Phys. Rev. **107**, 337 (1957).

[6] P. Reimann, *Foundation of Statistical Mechanics under Experimentally Realistic Conditions.* Phys. Rev. Lett. **101**, 190403 (2008).

[7] N. Linden, S. Popescu, A. J. Short, and A. Winter, *Quantum mechanical evolution towards thermal equilibrium.* Phys. Rev. E **79**, 061103 (2009).

[8] A. J. Short and T. C. Farrelly, *Quantum equilibration in finite time.* New J. Phys. **14**, 013063 (2012).

[9] T. Farrelly, F.G.S.L. Brandao, M. Cramer, *Thermalization and Return to Equilibrium on Finite Quantum Lattice Systems.* Phys. Rev. Lett. **118**, 140601 (2017).

[10] K. Morita, *Theory of Reversible Computing.* Springer (2017).

[11] A. Yedidia and S. Aaronson, *A Relatively Small Turing Machine Whose Behavior Is Independent of Set Theory*, arXiv:1605.04343.

[12] S. Aaronson, *The busy beaver frontier*, https://www.scottaaronson.com/papers/bb.pdf.
